# Analysis of the Rumen Microbiome and Metabolome to Study the Effect of an Antimethanogenic Treatment Applied in Early Life of Kid Goats

**DOI:** 10.3389/fmicb.2018.02227

**Published:** 2018-10-09

**Authors:** Leticia Abecia, Gonzalo Martínez-Fernandez, Kate Waddams, Antonio Ignacio Martín-García, Eric Pinloche, Christopher J. Creevey, Stuart Edward Denman, Charles James Newbold, David R. Yáñez-Ruiz

**Affiliations:** ^1^Estación Experimental del Zaidín, Consejo Superior de Investigaciones Científicas, Granada, Spain; ^2^Commonwealth Scientific and Industrial Research Organisation, Agriculture and Food, Queensland Bioscience Precinct, St Lucia, QLD, Australia; ^3^Institute of Biological, Environmental & Rural Sciences (IBERS), Aberystwyth University, Aberystwyth, United Kingdom; ^4^Institute for Global Food Security, Queen's University Belfast, Belfast, United Kingdom; ^5^Scotland's Rural College (SRUC), Edinburgh, United Kingdom

**Keywords:** rumen, early life, methane, metabolome, bromochloromethane

## Abstract

This work aimed to gain insight into the transition from milk to solid feeding at weaning combining genomics and metabolomics on rumen contents from goat kids treated with a methanogenic inhibitor (bromochloromethane, BCM). Sixteen goats giving birth to two kids were used. Eight does were treated (D+) with BCM after giving birth and over 2 months. One kid per doe in both groups was treated with BCM (k+) for 3 months while the other was untreated (k–). Rumen samples were collected from kids at weaning (W) and 1 (W + 1) and 4 (W + 4) months after and from does at weaning and subjected to 16S pyrosequencing and metabolomics analyses combining GC/LC-MS. Results from pyrosequencing showed a clear effect of age of kids, with more diverse bacterial community as solid feed becomes more important after weaning. A number of specific OTUs were significantly different as a result of BCM treatment of the kid at W while at W + 1 and W + 4 less OTUs were significantly changed. At W + 1, *Prevotella* was increased and *Butyrivibrio* decreased in BCM treated kids. At W + 4 only the effect of treating mothers resulted in significant changes in the abundance of some OTUs: *Ruminococcus, Butyrivibrio* and *Prevotella*. The analysis of the OTUs shared by different treatments revealed that kids at weaning had the largest number of unique OTUs compared with kids at W + 1 (137), W + 4 (238), and does (D) (23). D + k+ kids consistently shared more OTUs with mothers than the other three groups at the three sampling times. The metalobomic study identified 473 different metabolites. In does, lipid super pathway included the highest number of metabolites that were modified by BCM, while in kids all super-pathways were evenly affected. The metabolomic profile of samples from kids at W was different in composition as compared to W + 1 and W + 4, which may be directly ascribed to the process of rumen maturation and changes in the solid diet. This study shows the complexity of the bacterial community and metabolome in the rumen before weaning, which clearly differ from that after weaning and highlight the importance of the dam in transmitting the primary bacterial community after birth.

## Introduction

Ruminants are important for the conversion of feed resources that are not human edible into highly-valued, healthy human food (Gill et al., 2010). The major site for this conversion is the rumen where microbial fermentation of plant biomass produces up to 70% of the energy requirements of the host animal. The composition of rumen microbial population clearly influences fermentation efficiency, but also methane production (Morgavi et al., 2010). The release of methane (CH_4_) results in a loss of dietary energy (Johnson and Johnson, 1995) and once released into the environment, CH_4_ acts as a potent greenhouse gas, with a much greater effect on climate change than that of carbon dioxide (Intergovernmental Panel on Climate Change, 2006). Consequently, a better understanding of the microbial community within the rumen may facilitate the development of strategies to decrease the production of enteric CH_4_.

The microbial diversity in the rumen has been shown to depend very much on the diet (i.e., substrate to be fermented) and has a strong host specificity (Malmuthuge and Guan, 2017), which makes it difficult to achieve significant modulation in the adult animal once the rumen is fully developed and the microbial ecosystem established. The developing rumen provides an opportunity to explore means of microbial manipulation. The “proto-rumen” is first colonized by hydrogenotrophic acetogens, which are gradually replaced by methanogenic archaea as the rumen develops (Gagen et al., 2012). Studies in humans showed that early gut colonizers, such as those acquired from parents, can exert physiological, metabolic and immunological effects for most of our lives (Faith et al., 2013). Yáñez-Ruiz et al. (2010) observed that bacterial communities resulting from altering forage/concentrate ratio in early life persisted over 4 months. Imai et al. (2002) showed persistence of ciliates transferred from a deer to the rumen of a calf, whilst Gagen et al. (2012) suggested that early colonizing methanogens may persist in the rumen of adult animals.

Recently, we have shown (Abecia et al., 2013) that application of bromochloromethane (BCM) to goat kids modified archaeal colonization of the rumen, with the effects persisting for 3 months in kids raised by does that received the same treatment as the kids. This was further confirmed (Abecia et al., 2014a) by 454 pyrosequencing which showed different response of the archaeal community observed between offspring and adult goats, which may suggest that the competition occurring in the developing rumen to occupy different niches offer potential for intervention. However, no information is available on to what extent the bacterial community is affected by such treatments in early life and the effects exerted on the main metabolic pathways.

While comprehensive metabolomics studies of certain human biofluids have been undertaken (Wishart et al., 2008, 2009; Psychogios et al., 2011), only a few studies have used modern metabolomics technologies to characterize the rumen metabolome. Some recent work explored effects of diet on the rumen fluid metabolome (Ametaj et al., 2010a,b), but the focus was on rumen digestive disorders and less attempts have been made to link deep metabolomics profile in the rumen with the production of methane. Saleem et al. (2013) established a rumen fluid metabolome data base (www.rumendb.ca) using experimental and literature data containing 246 metabolites. Recently, Artegoitia et al. (2016) provided a comprehensive insight into biochemical mechanisms that are associated with feed efficiency in growing steers. However, to date there is no characterization of the changes occurring in metabolic pathways during the development of the rumen and the link with the microbial composition of the ecosystem. Understanding the phylogenetic composition and functional potentials of the rumen microbial community of pre-ruminant animals could guide efforts in the design of nutritional strategies applied in early life of the animals with impact in the adulthood.

The aim of this study was to gain insight into the processes occurring during the development of the rumen by combining genomics and metabolomics on rumen digesta samples from goat kids treated with a methanogenic inhibitor (BCM) and to assess to what extent the effects persist later in life. The results from this trial on rumen fermentation and CH_4_ production have been previously published in Abecia et al. (2013).

## Materials and methods

All management and experimental procedures involving animals were carried out by trained personnel in strict accordance with the Spanish guidelines (RD 1201/2005 of 10th October 2005) for experimental animal protection at the Estación Experimental del Zaidín. Experimental protocols were approved (1st October 2010) by the Ethics Committee for Animal Research at the Animal Nutrition Unit.

### Animals, diets, and experimental design

Sixteen Murciano-Granadina goats (43 ± 1.7 kg BW) pregnant with two fetuses were acquired at 3 months of pregnancy, kept in individual pens (1.7 × 1.2 m) with free access to water and fed alfalfa hay *ad libitum* and a concentrate supplement 600 g/day twice a day (0900 and 1,500 h).

The experimental period commenced when does gave birth, which happened within a period of 2 weeks. After giving birth, each doe was randomly allocated to 1 of the 2 experimental groups: **D**+, treated daily with 3 mg/kg BW of BCM divided in two equal doses embedded in 10 g of ground oats in cellulose paper and sealed with molasses, and **D**–, as control non-treated-group but receiving only the 10 g of ground oats in cellulose paper and sealed with molasses. Bromochloromethane (99.5%; Aldrich 13,526-7) is a halogenated aliphatic hydrocarbon entrapped in an alpha-cyclodextrin matrix (Alfa Aesar GmbH & Co, A18092) (May et al., 1995). The BCM formulation was prepared as a dry white powder in 1–2 kg batches and contained 10−12% (wt/wt) BCM. The BCM treatment was given orally twice a d at feeding times (0900 and 1500 h) to does.

**Figure 1:**
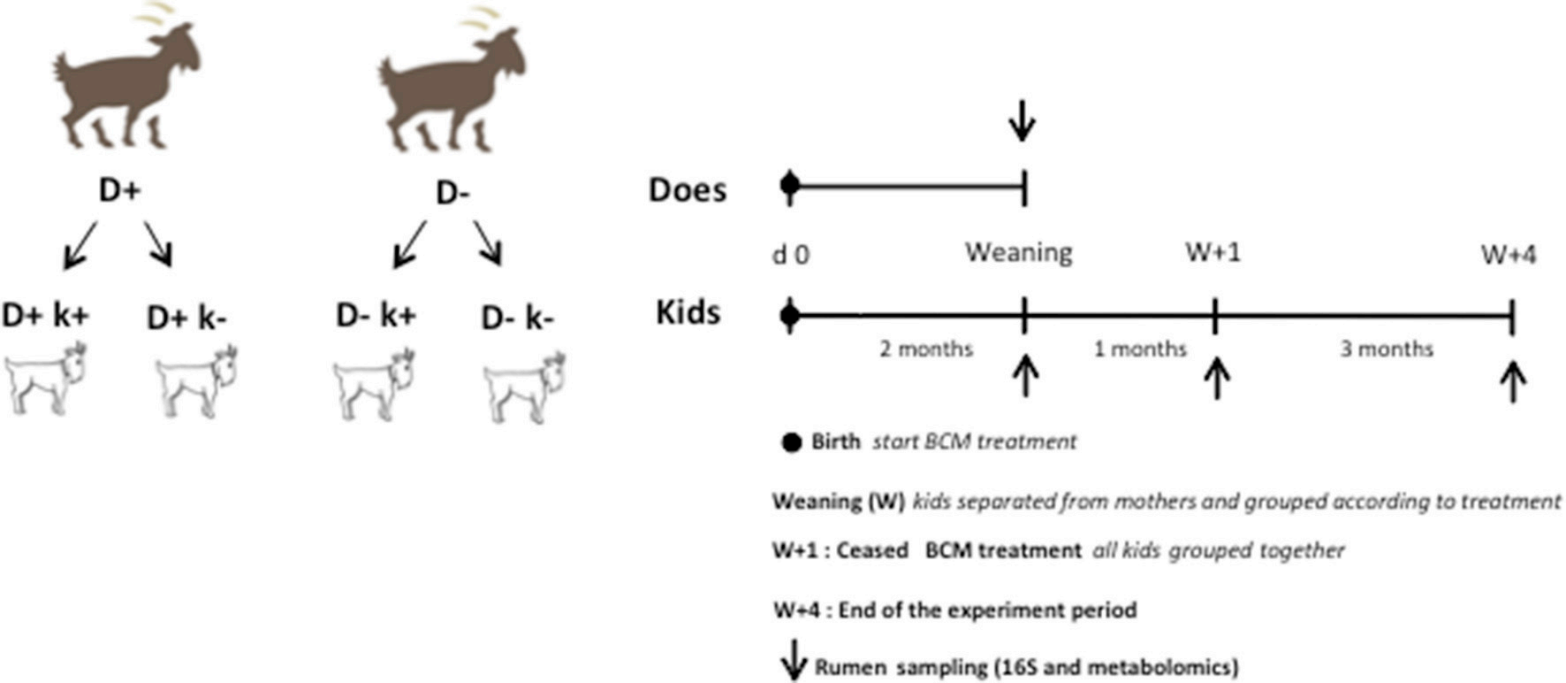
Experimental design and sampling schedule.

All does gave birth to 2 kids, one remained non-treated (**k**−) while the other was given a daily dose of 0.3 mg/kg BW of BCM as above (**k**+), thus resulting overall in four kids' experimental groups D + k+, D+k–, D–k+, D–k– (*n* = 8; Figure 1). During the first 2 weeks of life of the treated kids, the BCM formulation was directly inserted in the mouth of the animal dissolved in 10 ml of water twice a day. After 2 weeks, BCM treatment was given orally twice a d at feeding times (0900 h and 1500 h) to kids as described for does. The kids remained with does for 2 months in the same pen with no physical contact with other animals to avoid touching and licking. The treatment of kids lasted for 3: 2 months while they remained with the doe and for 1 months after weaning, during which kids were grouped by treatments (D + k+, D+k–, D–k+, D–k–) in 4 independent pens separated from each other to avoid physical contact. After weaning, kids were offered *ad libitum* alfalfa hay and starter commercial compound. At 3 months, all kids from the 4 experimental groups were grouped together in a single pen and BCM treatment ceased. They remained together for another 3 months until the end of the experimental period.

Ruminal content was collected at weaning from does and 3 times from kids: at weaning (W) and 1 (W + 1) and 4 months after (W + 4). Samples were taken before the morning feeding using a flexible PVC tube (2 mm of wall thickness and 5 mm of internal diameter; Cristallo Extra,FITT S.p.A., Sandrigo, Italy) with about 10 holes of 3 mm diameter in the probe head. The tube was warmed-up using hot water and inserted to a depth of approximately 120–150 cm via the esophagus. Rumen samples (ca. 10 ml) were obtained using an electric vacuum pump (down to 7 mbar; Vacuubrand MZ 2C, Wertheim, Germany). Aliquots were immediately stored at −80°C for further analyses (pyrosequencing and metabolomics).

### DNA extraction

Samples of rumen digesta were freeze-dried and thoroughly mixed by physical disruption using a bead beater (1 min at 5,000 rpm) (Mini-bead beater 8, BioSpec Products, Bartlesville, United States). The extraction of total DNA was performed from 50 mg samples using the QIAamp® DNA Stool Mini Kit (Qiagen Ltd, West Sussex, United Kingdom) following the manufacturer's instructions with a modification: a higher temperature (95°C) was used for lysis incubation. The yield and purity of the extracted DNA were assessed using NanoDrop® ND-1000 Spectrophotometer (NanoDrop Technologies, Wilmington, United States).

### PCR amplification of 16S

Amplification of the V1–V2 hyper-variable regions of 16S rRNA was carried out with primers 27F and 357R (Liu et al., 2007). The protocol followed for PCR amplification, short fragment removal and sequencing was described by Martínez-Fernández et al. (2015).

### Sequence preprocessing and statistical analysis

Following sequencing, data were combined and sample identification assigned to multiplexed reads using the UPARSE software environment (Edgar, 2013). Low quality sequences, pyrosequencing errors and chimeras were removed then sequences were clustered into operational taxonomic units (OTU's) at 97% identity. An OTU table with counts per sample was generated using UPARSE and imported into Phyloseq R package (1.23.1) (McMurdie and Holmes, 2013). Alpha diversity measures for samples grouped by collection period and treatments were performed on unrarefied data using Physoleq. The OTU count data was log transformed as an approximate variance stabilization transformation process prior to beta diversity analysis. Beta diversity was performed as a principal coordinate analysis for the Bray Curtis dissimilarity distances for each sample using Phyloseq. The significances of grouping in the PCoA plots were tested by analysis of dissimilarity (ADONIS) with 999 permutations from the vegan package (Oksanen et al., 2017). Venn diagrams illustrating the overlap of OTUs between groups were generated in R using VennDiagram (1.6.20) (Chen, 2018). Identification of OTUs significantly different between treatments represented as log2 fold changes for OTUs with adjusted *p* < 0.05 (false discovery rate) were calculated in the DESeq2 package (1.18.1) (Love et al., 2014). Plots were produced using the ggplot2 package (2.2.1) (Wickham, 2016). The effect of treatment groups across time and their interaction were calculated for alpha diversity and changes in taxonomic group relative abundances, with the animal as the experimental unit using the linear mixed model from the lme4 package (Bates et al., 2015). The sequences obtained have been deposited in the European Nucleotide Archive (ENA) under the accession number PRJEB27748.

### Metabolomics

A total of 76 freeze-dried rumen samples were sent to Metabolon (http://www.metabolon.com), 16 samples from does at weaning (8 treated and 8 untreated with BCM) and 5 samples per experimental group of kids (D + k+, D+k–, D–k+, D–k–) and collection period (W, W + 1, and W + 4). At the time of analysis samples were extracted and prepared for analysis using Metabolon's standard solvent extraction method (Sreekumar et al., 2009). The sample preparation process was carried out using the automated MicroLab STAR® system from Hamilton Company. Recovery standards were added prior to the first step in the extraction process for QC purposes. Sample preparation was conducted using a proprietary series of organic and aqueous extractions to remove the protein fraction while allowing maximum recovery of small molecules. The resulting extract was divided into two fractions; one for analysis by LC and one for analysis by GC. Samples were placed briefly on a TurboVap® (Zymark) to remove the organic solvent. Each sample was then frozen and dried under vacuum. Samples were then prepared for the appropriate instrument; either LC/MS or GC/MS. Compounds were identified by comparison to library entries of purified standards or recurrent unknown entities. Identification of known chemical entities was based on comparison to metabolomic library entries of purified standards. At the time of analysis, more than 1,000 commercially available purified standard compounds had been acquired registered into LIMS for distribution to both the LC and GC platforms for determination of their analytical characteristics. The combination of chromatographic properties and mass spectra gave an indication of a match to the specific compound or an isobaric entity. Additional entities could be identified by virtue of their recurrent nature (both chromatographic and mass spectral). A data normalization step was performed to correct variation resulting from instrument inter-day tuning differences. Essentially, each compound was corrected in run-day blocks by registering the medians to equal one (1.00) and normalizing each data point proportionately. The SPSS software (IBM Corp. Released 2015. IBM SPSS Statistics for Windows, Version 23.0. Armonk, NY, United States) was used for statistical analysis. Statistical significant differences among treatments were determined performing the Welch's two tailed *t*-test. An estimate of the false discovery rate (*q*-value) was calculated to take into account the multiple comparisons that normally occur in metabolomic-based by using the Benjamini–Hochberg method. All comparisons with a false discovery rate <0.1 were considered significantly different throughout the analysis.

Partial Spearman rank correlation tests were used to look for potential associations between the abundances of OTUs and metabolites. No significant results were observed and therefore data are not presented.

## Results

### Bacterial community

Microbial profiling analysis was performed using amplicon sequence data from the 16S rRNA gene. Quality filtering of raw sequencing reads produced on average 8,800 sequences per sample.

**Figure 2:**
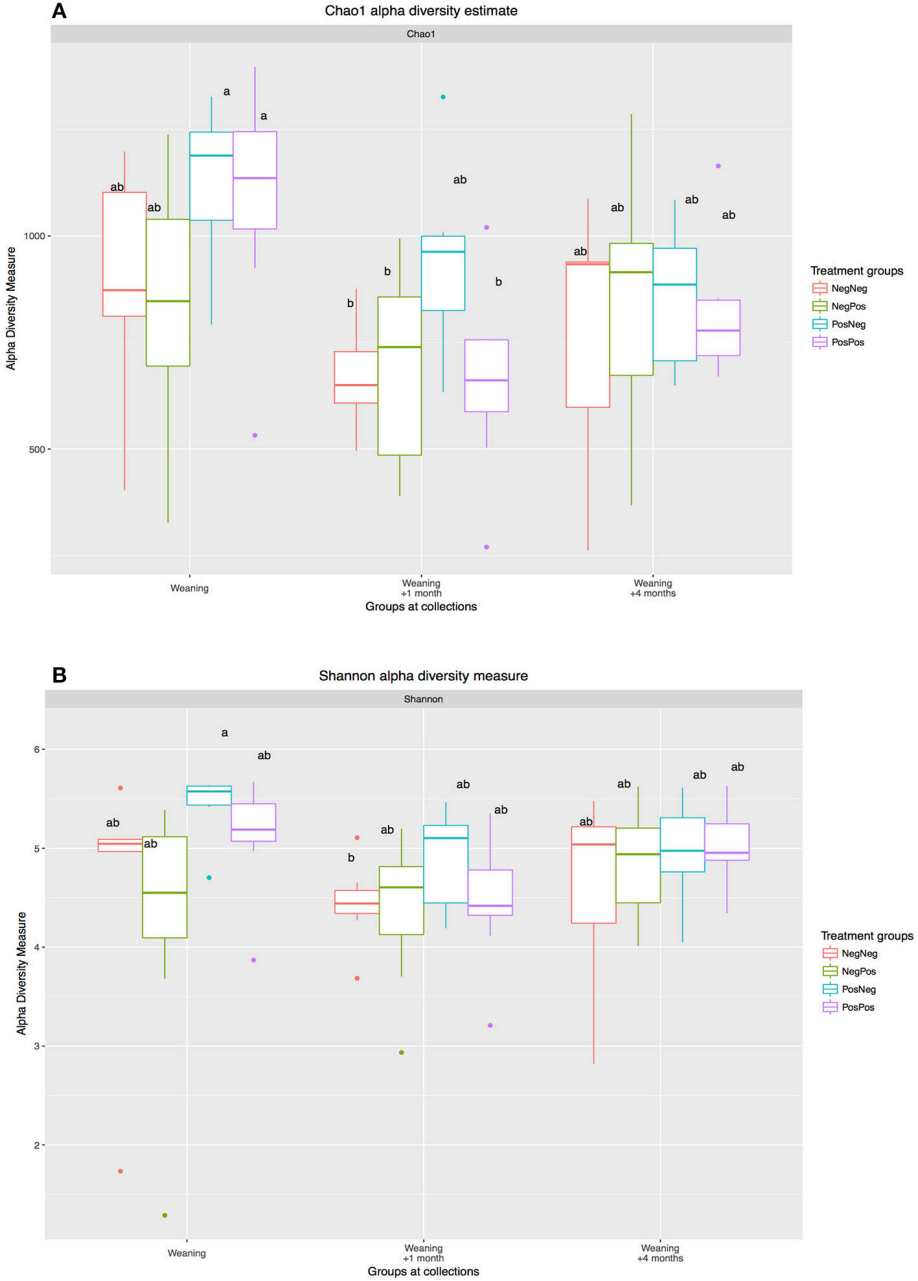
Alpha diversity measures: **(A)** Chao1 taxonomic units estimates and **(B)** Shannon diversity index): W, W + 1, and W + 4). Treatment groups: D–k–= NegNeg, D–k+ =NegPos, D+k– = PosNeg, D + k+ =PosPos. ^a,b^Letters denote significant differences between groups, bars that do not share the same letter are significantly different from each other (*P* < 0.05).

The analysis of the rumen bacteria community showed a highly diverse population in kids at W, with similar diversity indexes as in older animals (1 and 4 months after weaning, Figure 2). The treatment with BCM to mothers induced higher alpha diversity indexes in kids at W; however, these differences were no longer observed at W + 4.

**Figure 3:**
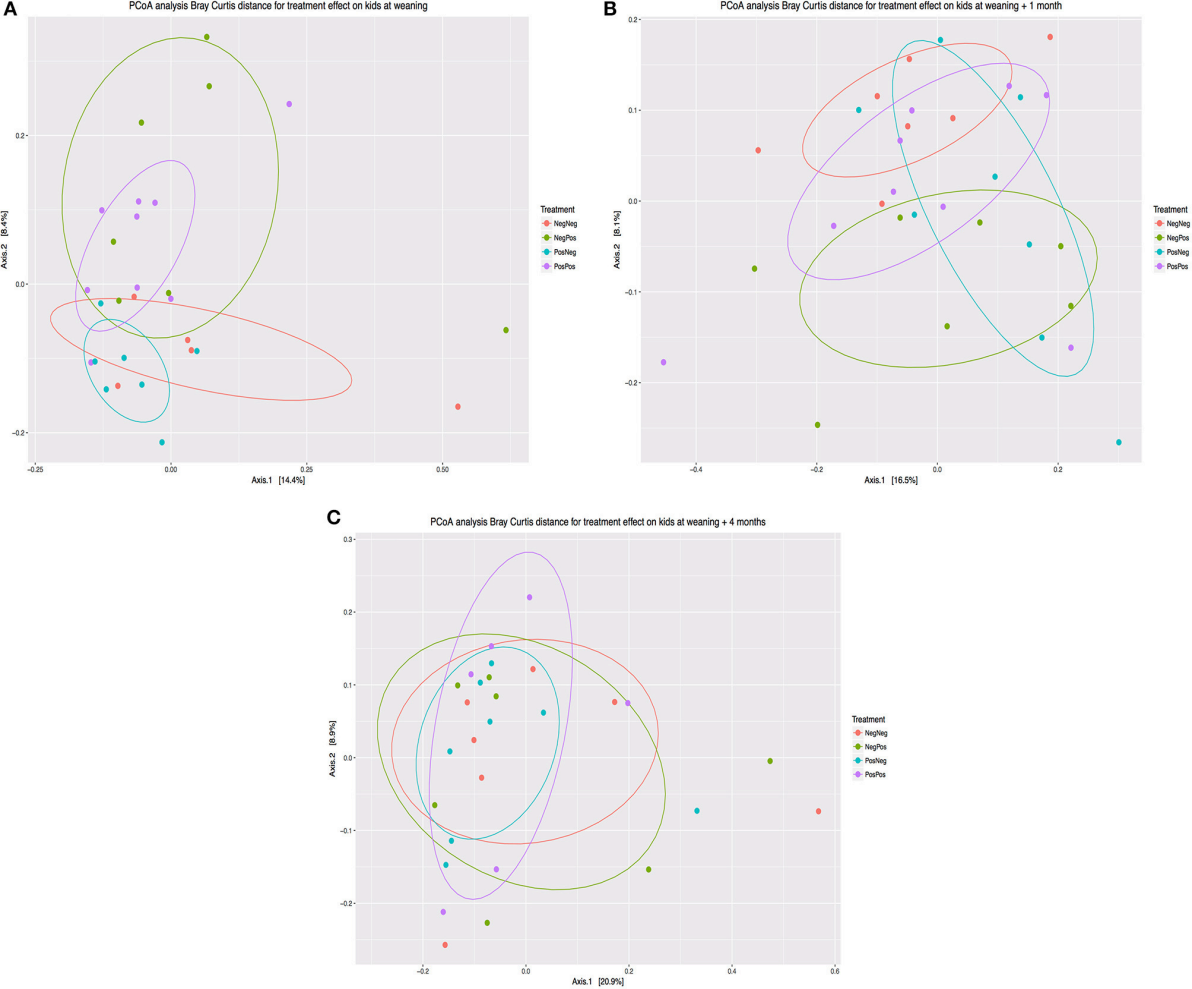
Principal coordinate analysis (Bray Curtis distance) comparing changes in rumen bacterial community at different times: **(A)** Weaning (W), **(B)** Weaning + 1 month (W + 1), **(C)** Weaning + 4 months (W + 4). Treatment groups: D–k– = NegNeg, D–k+ = NegPos, D+k– = PosNeg, D + k+ = PosPos.

The composition of the bacterial community determined by beta diversity analysis (Bray-Curtis) and multivariate analysis of variance (adonis) showed (Figure 3) statistical differences between groups at weaning (*P* = 0.015) and 1 month after (*P* = 0.007). However, no differences were observed at W + 4 (*P* = 0.894).

**Figure 4:**
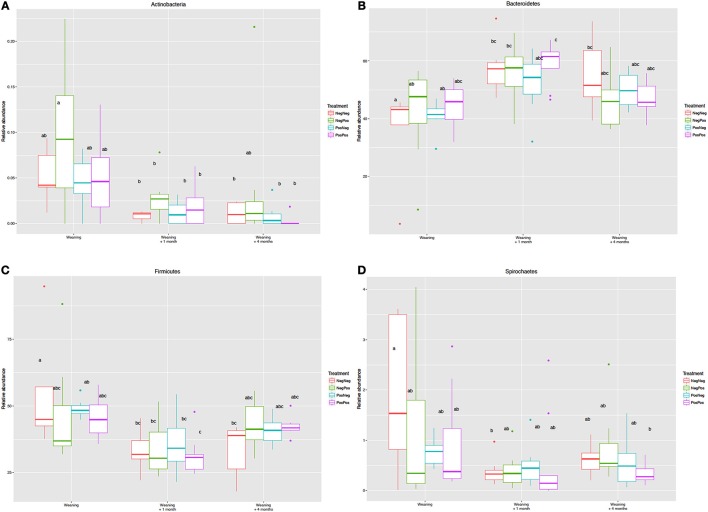
Relative abundance of the Phyla **(A)** Actinobacteria, **(B)** Bacteroidetes, **(C)** Firmicutes and **(D)** Spirochaetes in the different experimental groups at Weaning (W), Weaning + 1 month (W + 1), and Weaning + 4 months (W + 4) time points. Treatment groups: D–k– = NegNeg, D–k+ = NegPos, D+k– = PosNeg, D + k+ = PosPos. ^a,b^Letters denote significant differences between groups, bars that do not share the same letter are significantly different from each other (*P* < 0.05).

The analysis of the relative abundances of different phyla showed that the age of the animal and transition in diet rather than BCM treatment were the factors that caused most changes. Higher number of sequences assigned to Actinobacteria, Spirochaetes, Firmicutes, TM7, Tenericutes and Elusimicrobia and lower of Bacteroidetes were observed in animals at W compared to W + 1 and W + 4 (Figure 4).

At family level (Supplementary Figure 1), the abundance of Prevotellaceae was significantly (*P* < 0.05) lower in kids at W compared with W + 1 and W + 4. The sequences assigned to Bacteroidaceae were higher and Veillonellaceae were lower in D–k+ at W, and those from Ruminococcaceae and Dehalobacteriaceae were greater in kids non-receiving BCM at W. The group D–k– presented higher relative abundance of Actinomycetaceae, Lactobacillaceae and Planococcaceae at W. However, Anaeroplasmataceae, Clostridiaceaea and F16 families showed higher levels in D + k+ group at W compared to W + 1.

At the genus level (Supplementary Figure 2), the abundance of *Prevotella* was lower in all groups at W compared to older animals. At that age*, Parascardovia, Planomicrobium* and *Ruminococcus* were higher in D–k– group compared to W + 1 and W + 4 and other experimental groups. The abundance of *L7A_E11* increased and *Selenomonas* decreased in D+k+ compared to W + 1, however, *YRC22* abundance decreased compared to W + 4. In D–k+ group, *Bacteroides, BF311* and *Oscillospira* increased. *Dehalobacterium* abundance increased in D+k− group at W compare to W + 4.

**Figure 5:**
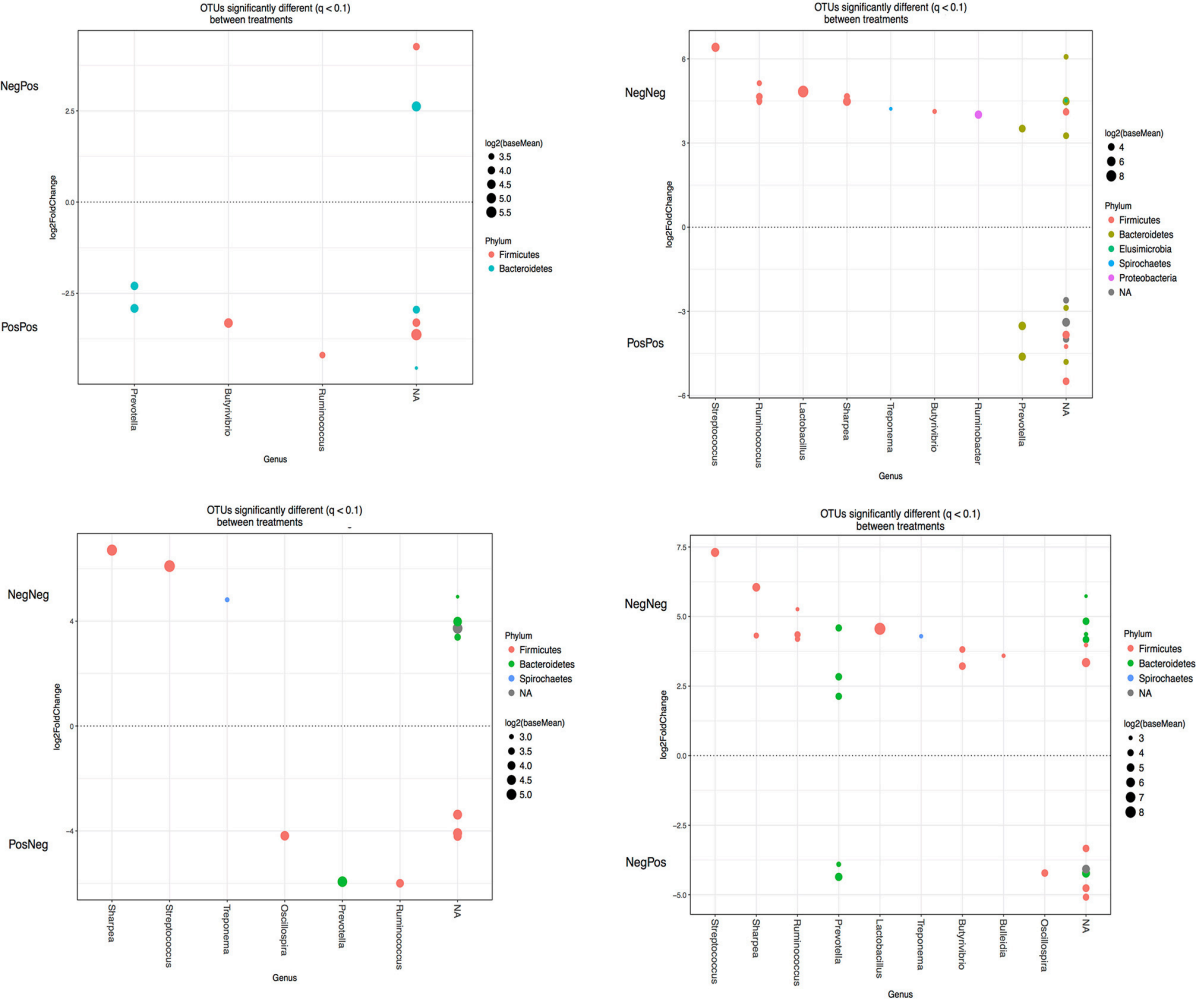
OTUs significantly different (*q* > 0.05 FDR) between different experimental groups at weaning. Upper and lower y-axis represents OTUs with a log2 fold positive and negative, respectively, difference for the experimental group treatment indicated above relative to treatment below. Each point represents a single OTU colored by phylum and grouped on the x axis by taxonomic genus level, size of point reflects the log2 mean abundance of the sequence data. Treatment groups: D–k– = NegNeg, D–k+ = NegPos, D+k– = PosNeg, D + k+ = PosPos.

A number of specific OTUs were significantly different (Figure 5) as a result of BCM treatment of the kid at W while at W + 1 and W + 4 less OTUs were significantly changed. At W + 1, *Prevotella* was increased and *Butyrivibrio* decreased in BCM treated kids. Interestingly, at W + 4 only the effect of treating mothers resulted in significant changes in the abundance of some OTUs: *Ruminococcus, Butyrivibrio* and *Prevotella* (data not shown).

**Figure 6:**
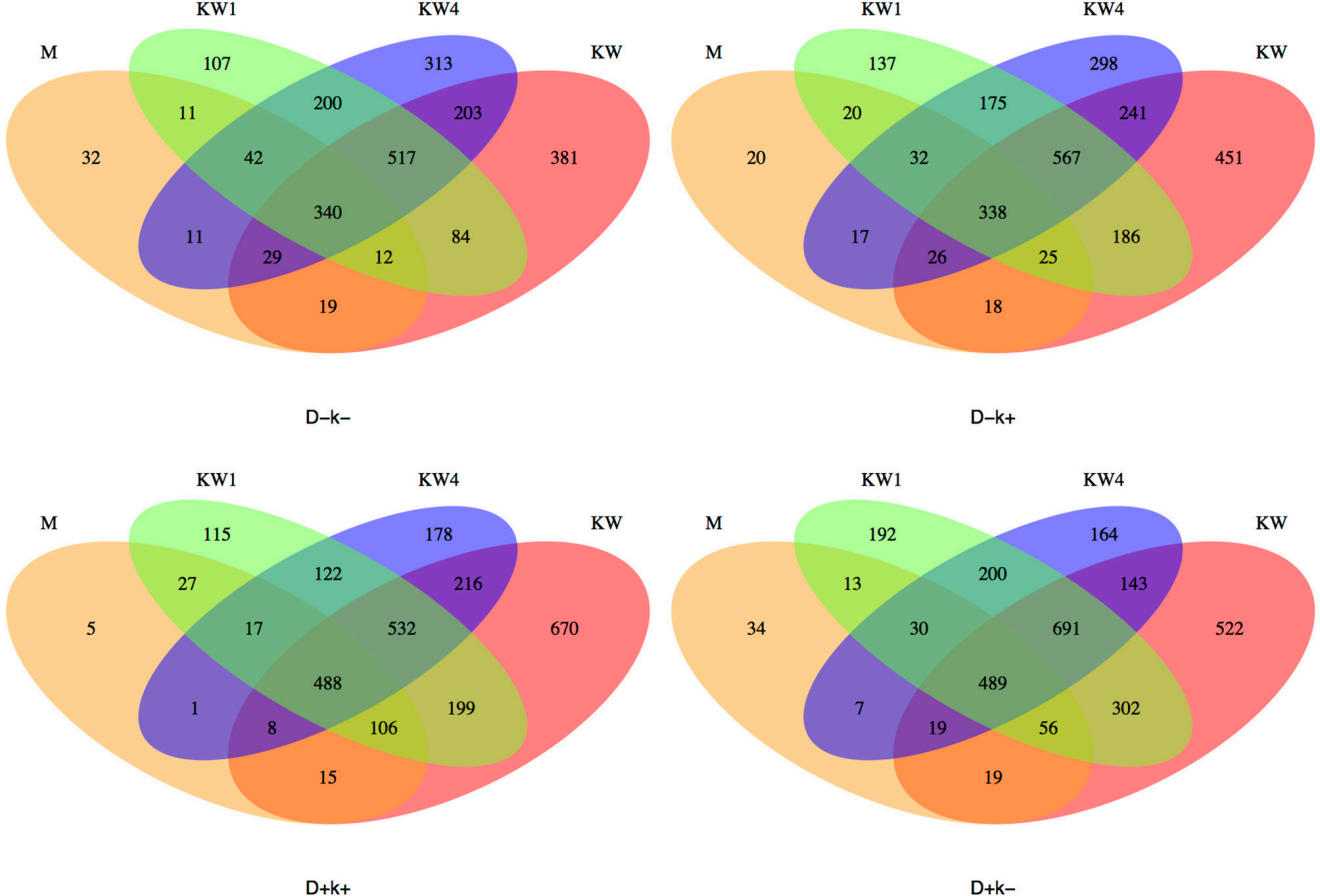
Venn plots showing the OTUs across different times (W, W + 1, and W + 4) in kids (K) and mothers (M) for each experimental group: D–k–, D–k+, D+k–, D + k+.

The analysis of the OTUs shared by different treatments (Figure 6) revealed that kids at weaning (W) had the largest number of unique OTUs (average across treatments 506) compared to W + 1 (137), W + 4 (238) and mothers (M) (23). Interestingly, D + k+ kids consistently shared more OTUs (as % of total) with mothers than the other three groups at the three sampling times.

### Rumen metabolomics profiling

Overall 473 metabolites were identified. Following log transformation and imputation with minimum observed values for each compound, Welch's two-sample *t*-tests were used to identify metabolites that differed significantly between positive and negative experimental groups (D+/D– and k+/k–) (Table 1). All super-pathways (amino acid, peptide, carbohydrate, lipid, nucleotide, cofactors and vitamins and xenobiotics) were affected, although to different extent depending on animals' age: 17 different metabolites in does, 19 in kids at W, 35 at W + 1 and 23 at W + 4. In does, lipid super pathway included the highest number of metabolites that were modified by BCM treatment (70% of total), while in kids all super-pathways were evenly affected. Most of the differences observed between BCM treated and non-treated kids across sampling times did not match differences between treated and non-treated mothers.

**Table 1:** Statistically significant (*p* < 0.10) biochemicals profiled in this study.

**Super pathway**	**Sub pathway**	**Biochemical name**		**W**							**W** + **1**							**W** + **4**							**Does**
				**1**	**2**	**3**	**4**	**5**	**6**		**1**	**2**	**3**	**4**	**5**	**6**		**1**	**2**	**3**	**4**	**5**	**6**		**D-/D+**
Amino acid	Glycine, serine and threonine	N-acetylthreonine																							
	Alanine and aspartate metabolism	alanine																							
	Glutamate metabolism	carboxyethyl-GABA																							
	Histidine metabolism	histidine																							
	Lysine metabolism	cadaverine																							
		glutarate (pentanedioate)																							
		N2-acetyllysine																							
	Phenylalanine & tyrosine metabolism	phenethylamine (isobar with 1-phenylethanamine)																							
		p-cresol sulfate																							
		phenylacetylglycine																							
	Tryptophan metabolism	2-aminophenol																							
	Valine, leucine and isoleucine	3-methyl-2-oxobutyrate																							
		3-methyl-2-oxovalerate																							
		levulinate (4-oxovalerate)																							
		2-hydroxyisobutyrate																							
	Cysteine, methionine, SAM, taurine	alpha-ketobutyrate																							
	Butanoate metabolism	2-aminobutyrate																							
	Polyamine metabolism	agmatine																							
		spermine																							
Peptide	Dipeptide	alanylalanine																							
		alanylglycine																							
		alanylphenylalanine																							
		aspartylphenylalanine																							
		phenylalanylalanine																							
		serylphenyalanine																							
	Gamma-glutamyl	gamma-glutamylvaline																							
		gamma-glutamylleucine																							
		gamma-glutamylisoleucine																							
		gamma-glutamylphenylalanine																							
Carbohydrate	Fructose, mannose, starch, sucrose	N-acetylmuramate																							
	Glycolysis, gluconeogenesis, pyruvate	glycerate																							
	Nucleotide sugars, pentose	ribulose																							
		isobar: ribulose 5-phosphate, xylulose 5-phosphate																							
Energy	Krebs cycle	alpha-ketoglutarate																							
Lipid	Essential fatty acid	linolenate 18:3n3																							
	Medium chain fatty acid	caprylate (8:0)																							
		pelargonate (9:0)																							
		caprate (10:0)																							
		undecanoate (11:0)																							
	Long chain fatty acid	oleate (18:1n9)																							
		nonadecanoate (19:0)																							
	Fatty acid, dicarboxylate	4-hydroxy-2-oxoglutaric acid																							
		suberate (octanedioate)																							
	Fatty acid, branched	13-methylmyristic acid																							
	Fatty acid metabolism	isovalerate																							
	Carnitine metabolism	deoxycarnitine																							
	Glycerolipid metabolism	glycerol 3-phosphate (G3P)																							
	Lysolipid	2-myristoylglycerophosphoethanolamine																							
	Monoacylglycerol	1-palmitoylglycerol (1-monopalmitin)																							
	Sterol	lathosterol																							
		squalene																							
		fucosterol																							
Nucleotide	Purine metabolism	xanthine																							
		xanthosine																							
		inosine																							
		inosine 5'-monophosphate (IMP)																							
	Purine metabolism, adenine	N6-methyladenosine																							
	Purine metabolism, guanine	guanine																							
		N2,N2-dimethylguanosine																							
	Pyrimidine metabolism, cytidine	cytidine 5'-monophosphate (5'-CMP)																							
	Pyrimidine metabolism, uracil	uracil																							
Cofactors and vitamins	Vitamin B6 metabolism	pyridoxate																							
Xenobiotics	Benzoate metabolism	3,4-dihydroxybenzoate																							
	Chemical	2-oxo-1-pyrrolidinepropionate																							
	Food component/Plant	coniferyl aldehyde																							
		enterolactone																							
		coumestrol																							
		2-oxindole-3-acetate																							
		1H-quinolin-2-one																							

*Red and green shaded cells indicate that the mean values are significantly higher or lower, respectively, for the pair-wise comparison (first treatment relative to second) as follows: (1) D–k–/D–k+, (2) D–k–**/**D+k+, (3) D–k–**/**D+k–, (4) D–k+**/**D+k–, (5) D–k+**/**D + k+, and (6) D+k–**/**D + k+. Kids (n = 5) and Does (n = 8)*.

The relevance of maternal influence was shown as hardly any difference was noted between D+k– and D+k– while a fair number of metabolites differed between D–k– and D+k– at W + 1 (12) and W + 4 (6). The most apparent long term effects of such maternal treatment on the metabolomics profile was observed in medium chain fatty acids (MCFA C6-C14), which were greatly elevated in the rumen of kids from mothers fed BCM compared to kids from mothers fed the control diet.

Given the difficulty of identifying a clear pattern of metabolomic profile changes along the sampling times for the different experimental groups, a heatmap for the major pathways was constructed including only those metabolites that were significantly changed in kids by BCM treatment at W, W + 1 and W + 4 (Supplementary Figure 3). The heatmap showed that samples taken at W were separated from those at W + 1 and W + 4, with no segregation of the latter two. In addition, a separated cluster group was observed at W corresponding to D-k+ kids.

## Discussion

In this study BCM was supplied to goats and kids to assess the impact of early life antimethanogenic treatment on the rumen bacterial community and metabolomics profile pre and after weaning and the potential persistency of effects after the treatment ceased. Bromochloromethane is a halogenated methane analog that affects methane production by reacting with reduced vitamin B12 and inhibiting the cobamide-dependent methyl transferase step of methanogenesis (Wood et al., 1968; Chalupa, 1977). This step is immediately before the terminal reductive reaction and is responsible for the synthesis of methyl coenzyme M (Wood et al., 1982). We have previously reported (Abecia et al., 2014a) that the application of BCM during early life of kids modified the archaeal community composition colonizing the rumen of kids, and that some less abundant archaeal groups remained different in treated and control animals 4 months after weaning.

It has been reported that microbial colonization of the developing rumen begins straight after birth and well before the animal achieves efficient consumption and digestion of solid feeds (Abecia et al., 2014b; Rey et al., 2014). At weaning, although the main bacterial groups are present (Rey et al., 2014), the anaerobic fermentation of plant material is still developing which is reflected in different microbial abundances as compared to adult animals in which rumen function is fully established (Jami et al., 2013). Thus, in kids at weaning Actinobacteria, Firmicutes and Elusinomicrobia presented greater abundances than in kids after weaning (W + 1 and W + 4), whilst that of Bacteroidetes was lower. These changes reflect the dietary transition from milk to solid diet given in production settings to adapt the animal to an adult feeding regimen (Abecia et al., 2014b, 2017). This is further confirmed by Wang et al. (2016) that compared the bacterial community in lambs at the age of weaning (42 days) who had received starter feed from d 7 as compared to those that only had access to milk. In accordance with our results lambs that were fed only milk showed greater abundances of Actinobacteria and lower of Bacteroidetes.

We hypothesize, that the likely effect of BCM on altering bacteria population is from changes in methane related metabolic pathways and H_2_ partial pressure (Mitsumori et al., 2012; Denman et al., 2015). A universal driving force in fermentation is to maximize ATP yield while disposing of hydrogen equivalents (Wolin et al., 1997). Thus, by maintaining a low partial pressure of H_2_, the methanogenic archaea indirectly change the flux of fermentation by bacteria, protozoa, and fungi that express hydrogenase to produce H_2_ while stoichiometrically increasing acetate and butyrate production. If this interspecies H_2_ transfer increases the ATP yield for some bacteria, it is expected that, in reverse, inhibition of methanogenic archaea by compounds such as BCM should thereby modify bacterial populations (Karnati et al., 2009). Mitsumori et al. (2012) observed using DGGE that BCM methane-inhibited rumen in adult goats adapted to high H_2_ levels by shifting fermentation to propionate via an increase in *Prevotella spp*. The BCM treatment at weaning significantly decreased the abundance of several genera such as Ruminoccus and Lactobacillus within Firmicutes. The lower relative abundances of sequences assigned to fibrolytic bacteria Ruminococcus is in agreement with Mitsumori et al. (2012) and Martínez-Fernández et al. (2015) using similar methane inhibitors. Interestingly at W + 1, this effect was no longer observed and the major impact of BCM treatment was shown on OTUs belonging to Butyrivibrio. Within the Bacteroidetes group, sequences assigned to Prevotella were affected by BCM at W and at W + 1, which is also in agreement with Mitsumori et al. (2012) and Denman et al. (2015). This main change in bacterial relative abundances observed in previous works was also noted at weaning, even though methane production at that stage of life is low.

In this study the persistency of effects caused after BCM treatment ceased (W + 1) was assessed 3 months later (W + 4). We have previously reported that at W + 4 D+k+ kids produced less CH_4_ than D–k+ ones (Abecia et al., 2013) along with persistent differences in the archaeal community structure (Abecia et al., 2014a). In line with changes observed at W and W + 1, specific OTUs assigned to Ruminococcus, Butyrivibrio and Prevotella were significantly different between D + k+ and D–k+ kids. An interesting observation is that the medium term persistency of effects on bacterial groups abundance was associated with the treatment applied to the mothers and not only to kids. The maternal influence on the newborn's microbial colonization of the gut has been described previously, specifically in humans (Jašarević et al., 2015; Mueller et al., 2015). However, the impact in ruminants is less clear, especially in the long-term (Yáñez-Ruiz et al., 2015). Recently, Yeoman et al. (2018) reported that the microbiota in the gastrointestinal tract (GIT) of calves (from 1 to 21 days old) was highly influenced by microbes in the dam's vagina, udder skin and colostrum. All three maternal sources comprised a unique microbial reservoir that shared OTUs with all examined calf GIT locations that persisted through the 21 days sampling period. Despite the brevity of the dam-calf interaction in Yeoman et al. (2018) study (calves were separated shortly after birth), their results suggest a very early and long time persistent maternal imprint. In our study kids were in contact with dams until weaning, which brings the oral microbiota as another important source of inoculation. Ruminants frequently regurgitate digesta from the rumen in a process known as rumination, which could facilitate the oral transfer of important rumen microbial groups. The fact that D+k+ kids consistently shared more OTUs (as % of total) with their mothers than the other experimental groups (Figure 6) at the three sampling times suggests that an intervention applied to newborns will have a long term impact if it is also applied to the mothers since they are the main source of inoculation. This is likely occurring through buccal contact as buccal swab samples have been shown to contain a large subpopulation of ruminant microbiota (Kittelmann et al., 2015). This would, in part, explain the longer-term persistency of effects associated to mothers' treatment since they exert the effect at the time the microbial ecosystem is being developed. Studies following up dams and offspring for longer periods are needed to fully understand the impact of such maternal effect.

The complexity of the rumen microbial metabolome was shown by the high number of metabolites identified (476). Previous studies have detected a variable range of biochemicals in the rumen (Saleem et al., 2013; Artegoitia et al., 2016) but no information is available on ruminants prior and/or post weaning. The range of metabolites found in the rumen is mainly characterized by phospholipids, inorganic ions, gases, amino acids, dicarboxylic acids, fatty acids, volatile fatty acids, glycerides, carbohydrates and cholesterol esthers, some have microbial origin while others are plant derived components. Since samples used for the metabolomics analysis in this study were composed of freeze-dried whole rumen digesta, it is very likely that both microbial and plant metabolites contribute to the final profile.

The substrate to be fermented in the rumen determines the dominant microorganisms in the ecosystem (Henderson et al., 2015) and the subsequent derived metabolites profile (Zhao et al., 2014). As described above, at weaning the intake of solid feed is still limited and the rumen is not fully functionally developed in terms of contractions, rumination and therefore the residence time of digesta may differ from that in later stages (Heinrichs, 2005). The metabolomic profile of samples from kids at W was different in composition as compared to W + 1 and W + 4. This may be directly ascribed to the above-mentioned process of rumen maturation and changes in the pattern of solid feed intake (Rey et al., 2014), which mainly occurred between weaning and the following month and not so much after solid feed intake is fully established. Further support of this distinctive ecosystem pre and post-weaning is the fact that the metabolites that were significantly affected by BCM in kids at W and W + 1 were not the same. A total of 16 metabolites differed significantly in the rumen of kids at W; however, at W + 1 the number of metabolites affected were twice as much as at W (31), mainly as a result of more differences in peptides and amino acids metabolites, specifically in Gamma-glutamyl, Leucine, Isoleucine and Valine metabolism sub-pathways, which were more abundant. This is likely due to the increase in Prevotella abundance post weaning as more plant protein enters in the rumen and the positive effect of BCM addition on the numbers of Prevotella observed in this work and others (Mitsumori et al., 2012). Another group of metabolites that differed across groups at W + 1 and not so much at W was that including plant components (enterolactone, apigenin, pheophorbidede A, homostachydrine, coumesterol, 2-onindole-3-acetate, phytol and phytanate), which reinforces the idea that the greater intake of plant material at W + 1 (as compared to W) (Rey et al., 2014) is the key driving factor and that there may have been differences in the intake pattern between treatments. These plant components did not differ across treatments at W + 4.

Despite the expected impact of rumen maturation on metabolic profile, 10 biochemicals differed in abundance at W + 4 as a consequence of the BCM treatment applied to the mothers. The most apparent maternal-driven effect on the rumen biochemical composition of kids was observed in medium chain fatty acids: caprylate (8:0), pelargonate (9:0), caprate (10:0), undecanoate (11:0) and laurate (12:0), which were greatly elevated in the rumen of kids from mothers fed BCM compared to kids from mothers fed the control diet. In a previous work we reported that lactating goats treated with BCM exhibited greater concentrations of <16 carbon fatty acids in milk than control ones (Abecia et al., 2012). Since MCFA in milk derive from microbial production in the rumen, this suggests that the maternal influence is mediated through inoculation of key microbial groups during the suckling period (Yeoman et al., 2018).

In summary, the results of this study show the complexity of the bacterial community and metabolome in the rumen before weaning, which clearly differ from that after weaning when solid feed intake is fully established. They also highlight the importance of the dam in transmitting the primary bacterial community after birth and call for further investigation regarding their role to enable permanent changes in the offspring later in life.

## Author contributions

DY-R, LA, and CN conceived and designed the experiments and analytical approaches. LA, GM-F, DY-R, and AM-G performed the animal trial. LA, KW, and GM-F analyzed the biological samples. LA, GM-F, CC, EP, and SD analyzed the data. LA and DY-R wrote the manuscript. All authors agree to be accountable for all aspects of the work.

### Conflict of interest statement

The authors declare that the research was conducted in the absence of any commercial or financial relationships that could be construed as a potential conflict of interest.

## Supplementary material

Supplementary Figure 1 Heatmap for the major metabolic pathways constructed including metabolites that were significantly changed in kids by BCM treatment at W, W + 1, and W + 4.

Supplementary Figure 2 Relative abundance of some Families in the different experimental groups at Weaning (W), Weaning + 1 month (W + 1), and Weaning + 4 months (W + 4) time points. Treatment groups: D−k− = NegNeg, D−k+ = NegPos, D+k− Q14 = PosNeg, D+k+ = PosPos.

Supplementary Figure 3 Relative abundance of some Genera in the different experimental groups at Weaning (W), Weaning + 1 month (W + 1), and Weaning + 4 months (W + 4) time points. Treatment groups: D−k− = NegNeg, D−k+ = NegPos, D+k− = PosNeg, D+k+ = PosPos.
