# Supplementary figures and images for: Analysis of the Rumen Microbiome and Metabolome to Study the Effect of an Antimethanogenic Treatment Applied in Early Life of Kid Goats
Source: Front Microbiol. 2018 Oct 9;9:2227. doi: 10.3389/fmicb.2018.02227 (PMC6189281; doi:10.3389/fmicb.2018.02227)

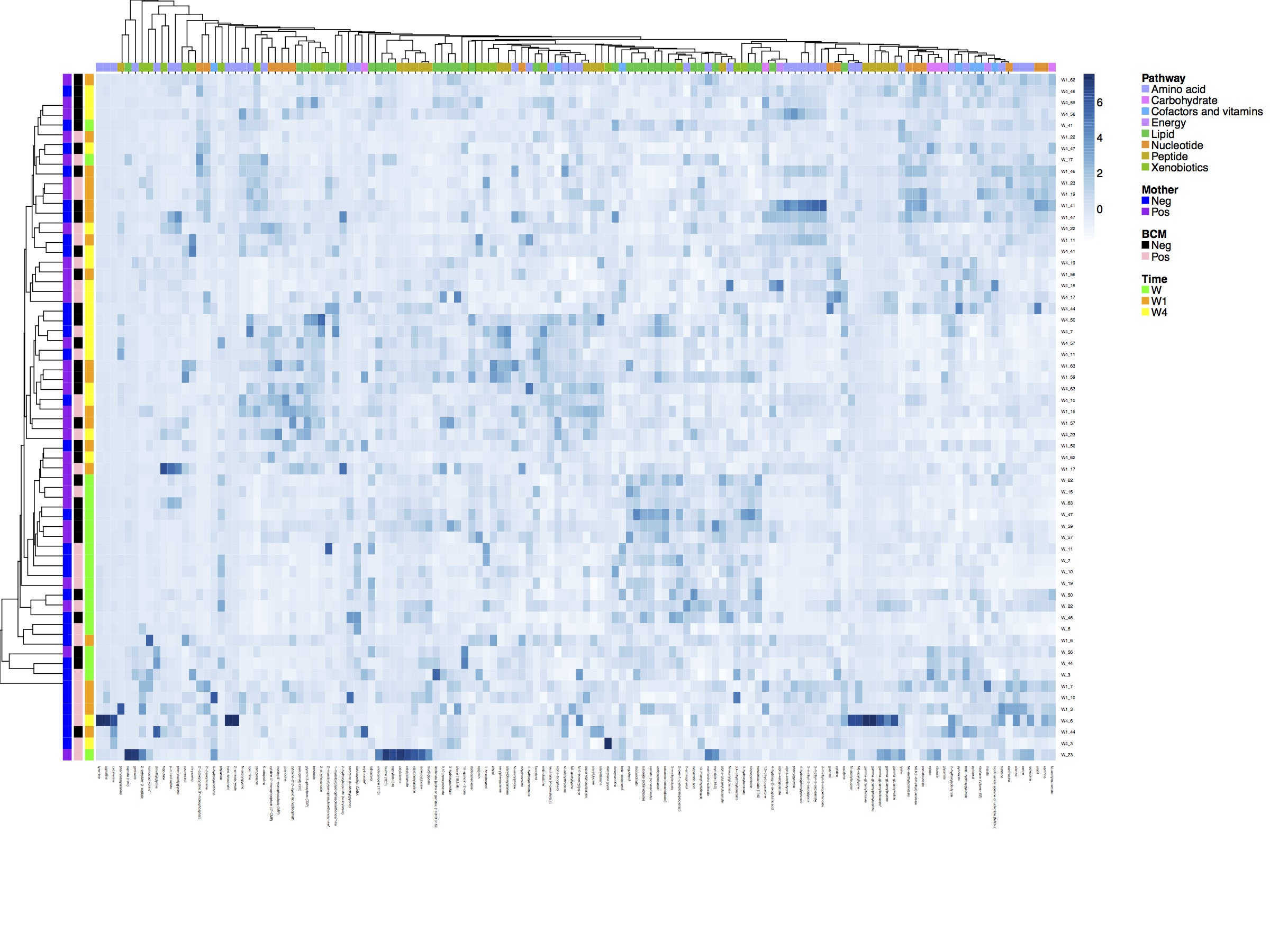

Supplement: Supplementary Figure 1 — Heatmap for the major metabolic pathways constructed including metabolites that were significantly changed in kids by BCM treatment at W, W + 1, and W + 4. [file Image_1.JPEG]
