# Supplementary material for: Analysis of the Rumen Microbiome and Metabolome to Study the Effect of an Antimethanogenic Treatment Applied in Early Life of Kid Goats
Source: Front Microbiol. 2018 Oct 9;9:2227. doi: 10.3389/fmicb.2018.02227 (PMC6189281; doi:10.3389/fmicb.2018.02227)
Supplement: Supplementary Figure 2 — Relative abundance of some Families in the different experimental groups at Weaning (W), Weaning + 1 month (W + 1), and Weaning + 4 months (W + 4) time points. Treatment groups: D−k− = NegNeg, D−k+ = NegPos, D+k− Q14 = PosNeg, D+k+ = PosPos. [file Image_2.pdf]

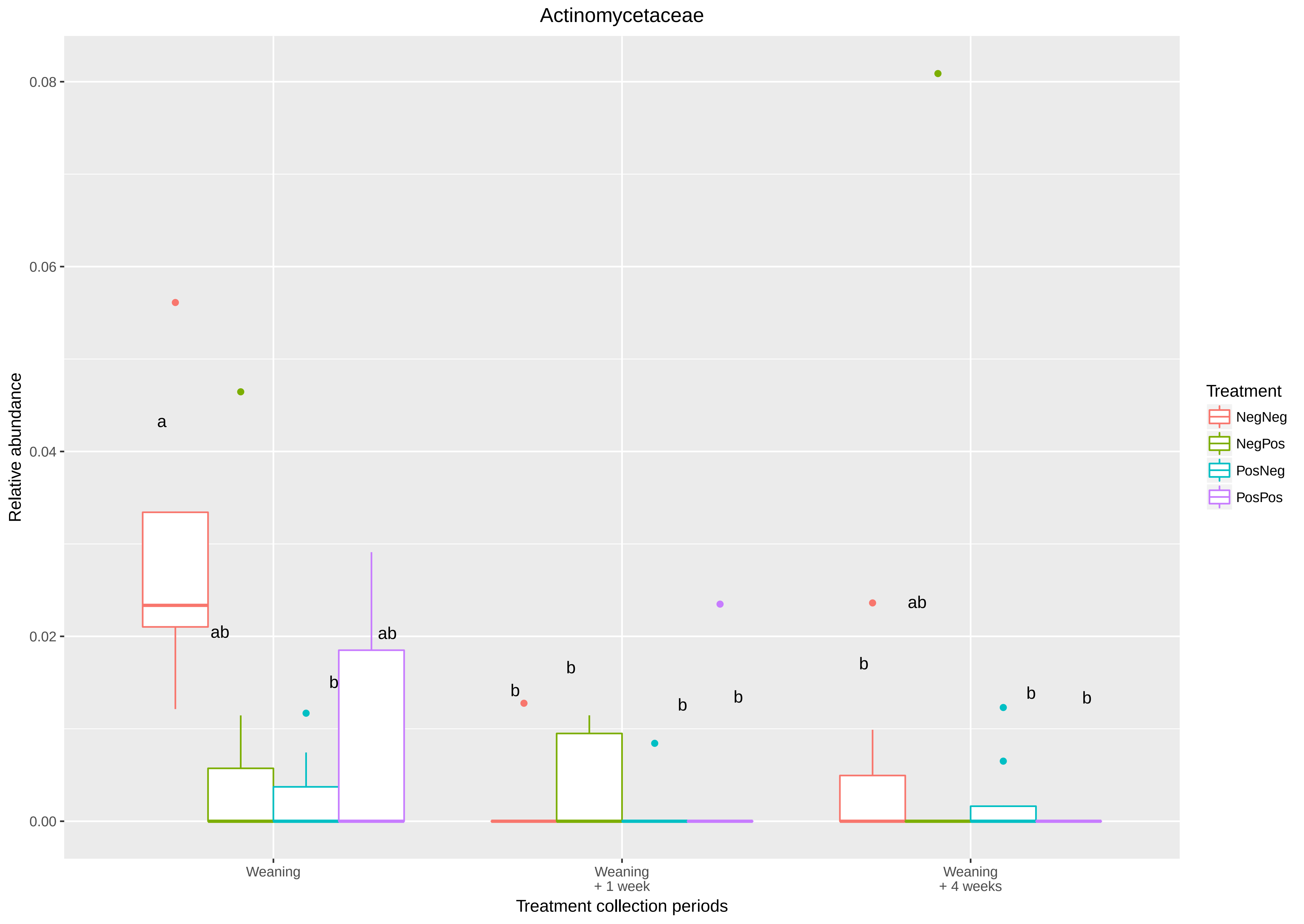

Actinomycetaceae
Relative abundance
0.08
0.06
0.04
0.02
0.00
a
ab
b
ab
b
b
b
b
ab
b
b
b
Weaning
Weaning
+ 1 week
Weaning
+ 4 weeks
Treatment collection periods
Treatment
NegNeg
NegPos
PosNeg
PosPos

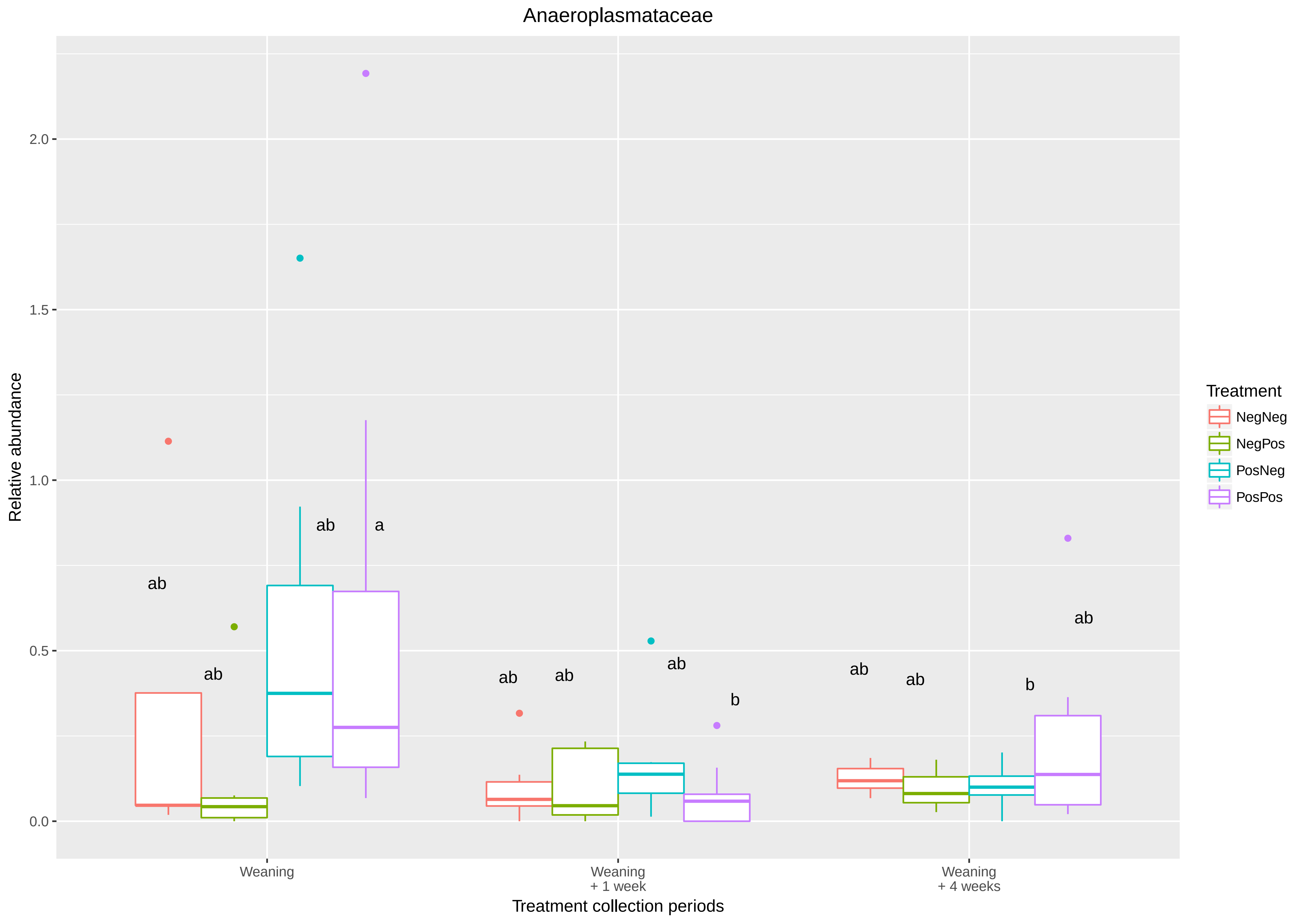
Anaeroplasmataceae
Relative abundance
0.0
0.5
1.0
1.5
2.0
ab
ab
ab
a
ab
ab
ab
b
ab
ab
b
ab
Weaning
Weaning
+ 1 week
Weaning
+ 4 weeks
Treatment collection periods
Treatment
NegNeg
NegPos
PosNeg
PosPos

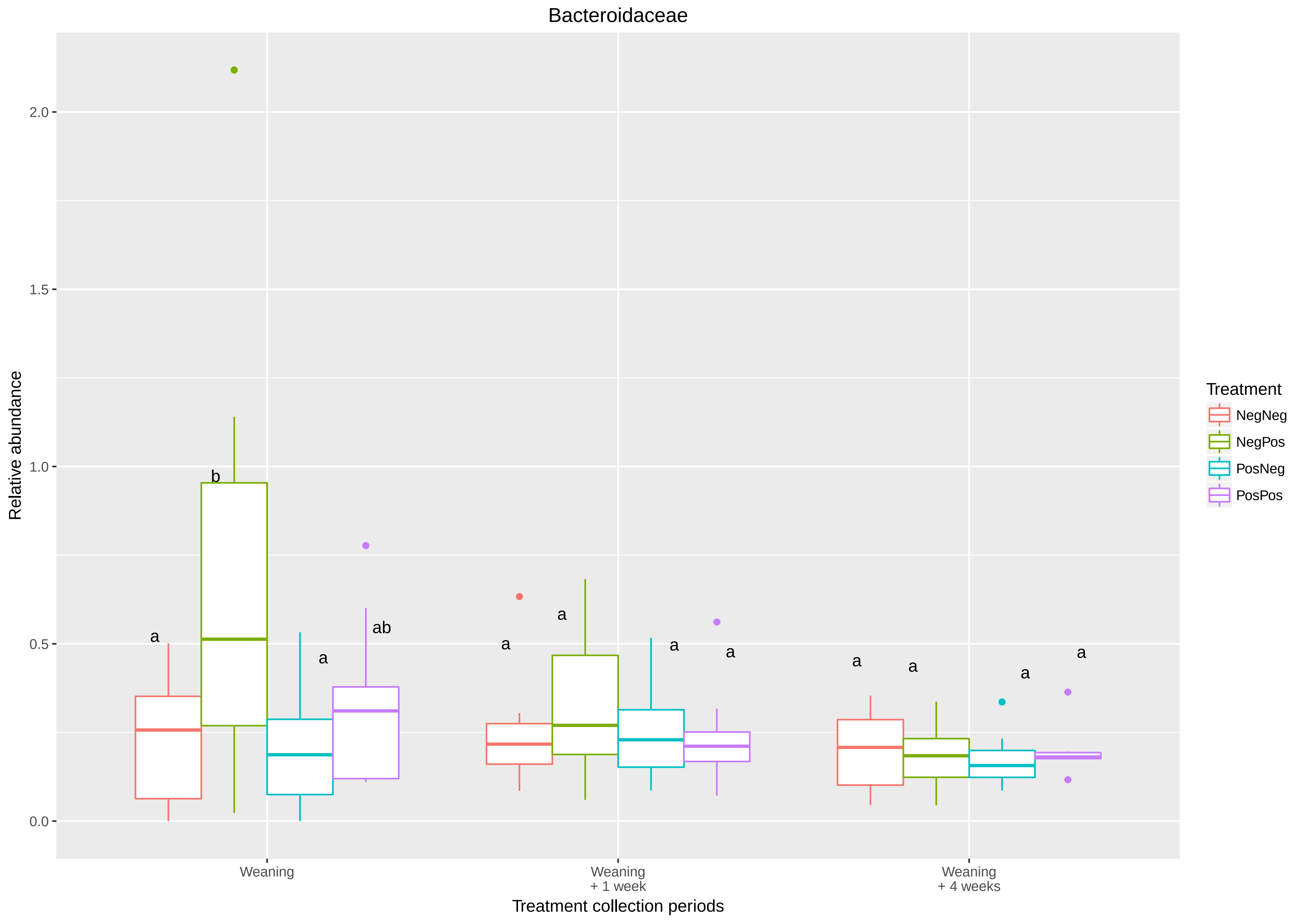
Bacteroidaceae
Relative abundance
2.0
1.5
1.0
0.5
0.0
b
a
a
ab
a
a
a
a
a
a
a
a
Weaning
Weaning
+ 1 week
Weaning
+ 4 weeks
Treatment collection periods
Treatment
NegNeg
NegPos
PosNeg
PosPos

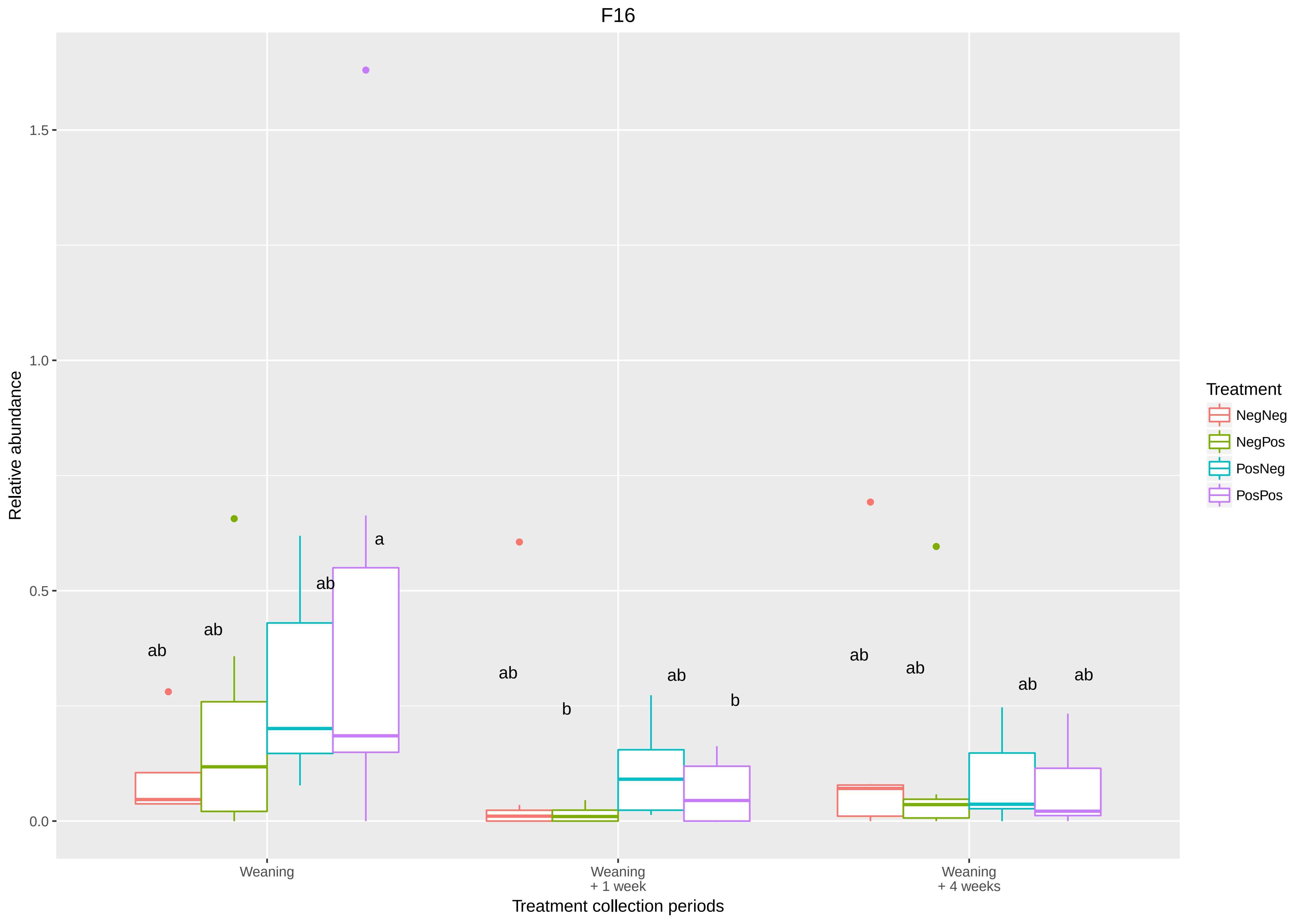
F16
Relative abundance
0.0
0.5
1.0
1.5
a
ab
ab
ab
ab
b
ab
b
ab
ab
ab
ab
Weaning
Weaning
+ 1 week
Weaning
+ 4 weeks
Treatment collection periods
Treatment
NegNeg
NegPos
PosNeg
PosPos

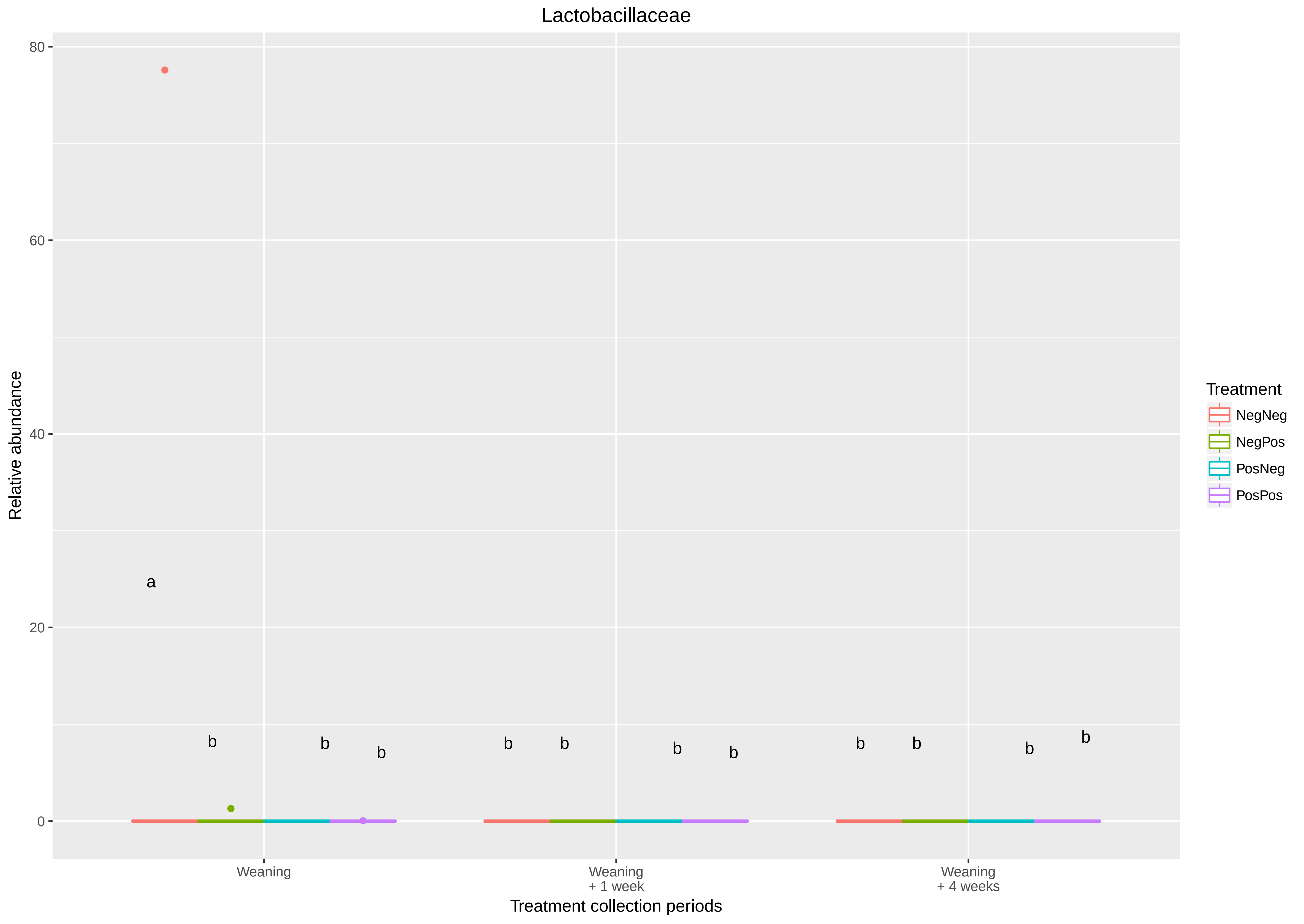

Lactobacillaceae
Relative abundance
80
60
40
20
0
a
b
b
b
b
b
b
b
b
b
b
b
Weaning
Weaning
+ 1 week
Weaning
+ 4 weeks
Treatment collection periods
Treatment
NegNeg
NegPos
PosNeg
PosPos

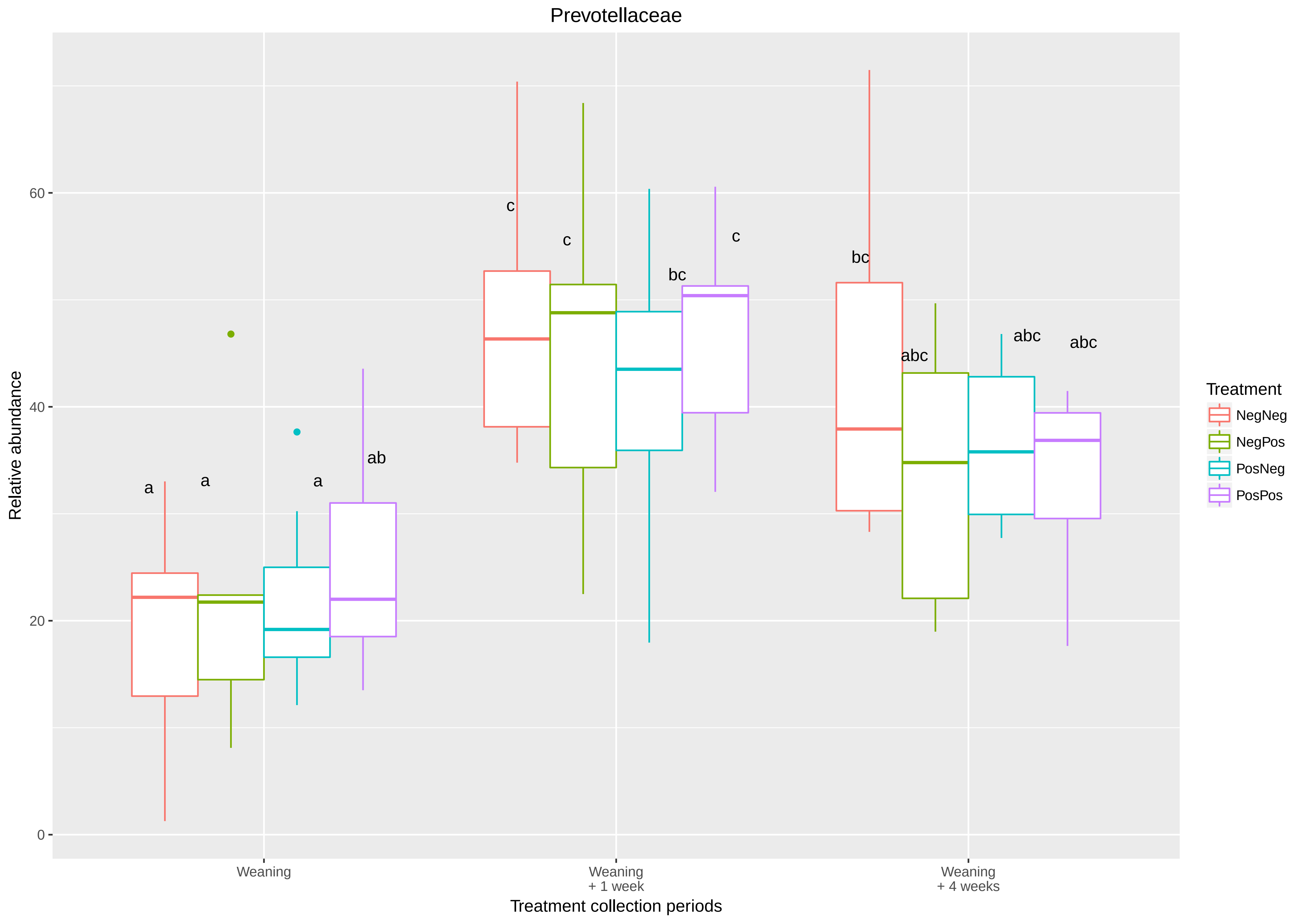

Prevotellaceae
Relative abundance
0
20
40
60
a
a
a
ab
c
c
bc
c
bc
abc
abc
abc
Weaning
Weaning
+ 1 week
Weaning
+ 4 weeks
Treatment collection periods
Treatment
NegNeg
NegPos
PosNeg
PosPos

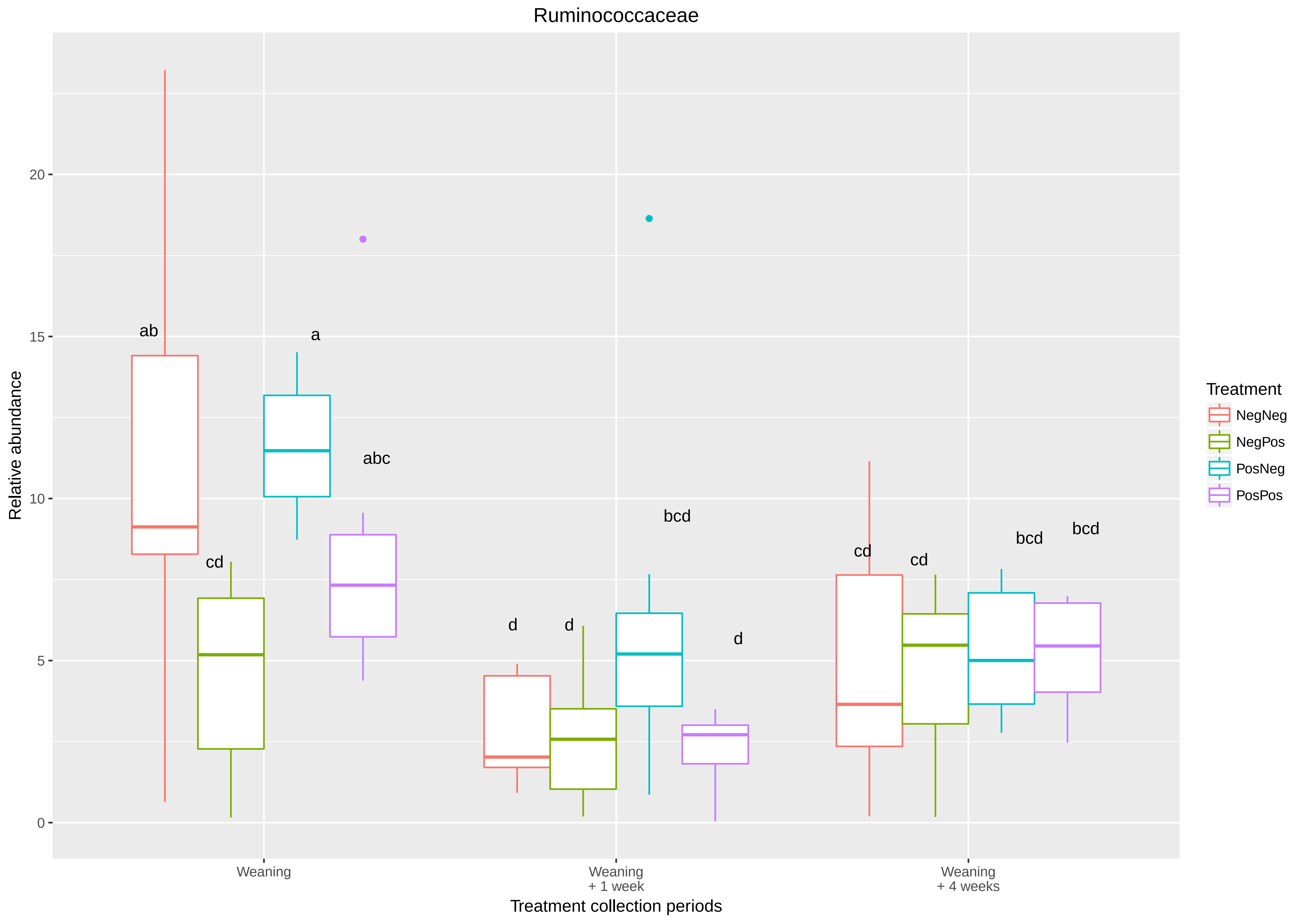
Ruminococcaceae
Relative abundance
0
5
10
15
20
ab
a
abc
cd
d
d
bcd
d
cd
cd
bcd
bcd
Weaning
Weaning
+ 1 week
Weaning
+ 4 weeks
Treatment collection periods
Treatment
NegNeg
NegPos
PosNeg
PosPos

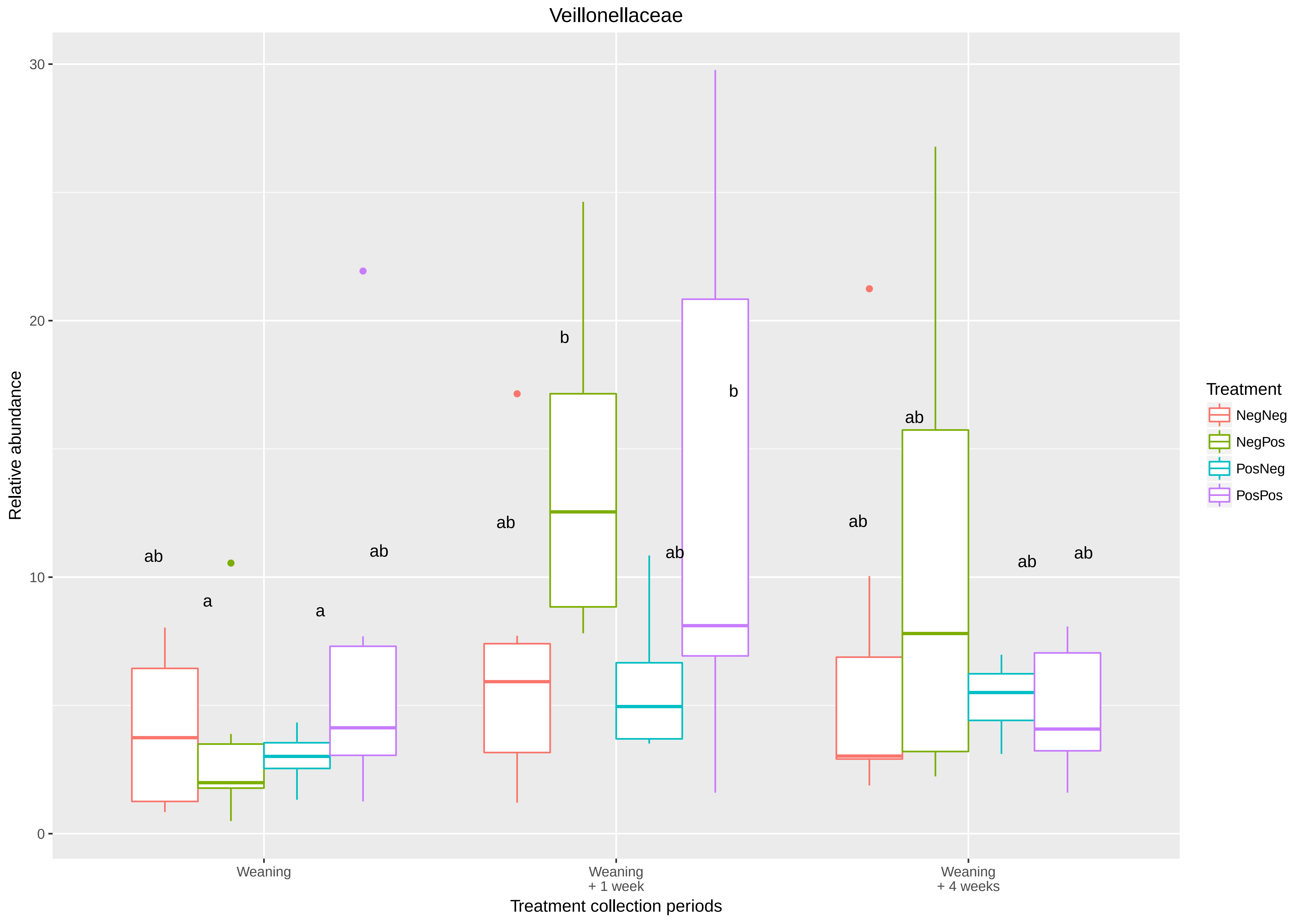

Veillonellaceae
Relative abundance
30
20
10
0
ab
a
a
ab
ab
b
ab
b
ab
ab
ab
ab
Weaning
Weaning
+ 1 week
Weaning
+ 4 weeks
Treatment collection periods
Treatment
NegNeg
NegPos
PosNeg
PosPos
