# Supplementary material for: Analysis of the Rumen Microbiome and Metabolome to Study the Effect of an Antimethanogenic Treatment Applied in Early Life of Kid Goats
Source: Front Microbiol. 2018 Oct 9;9:2227. doi: 10.3389/fmicb.2018.02227 (PMC6189281; doi:10.3389/fmicb.2018.02227)
Supplement: Supplementary Figure 3 — Relative abundance of some Genera in the different experimental groups at Weaning (W), Weaning + 1 month (W + 1), and Weaning + 4 months (W + 4) time points. Treatment groups: D−k− = NegNeg, D−k+ = NegPos, D+k− = PosNeg, D+k+ = PosPos. [file Image_3.pdf]

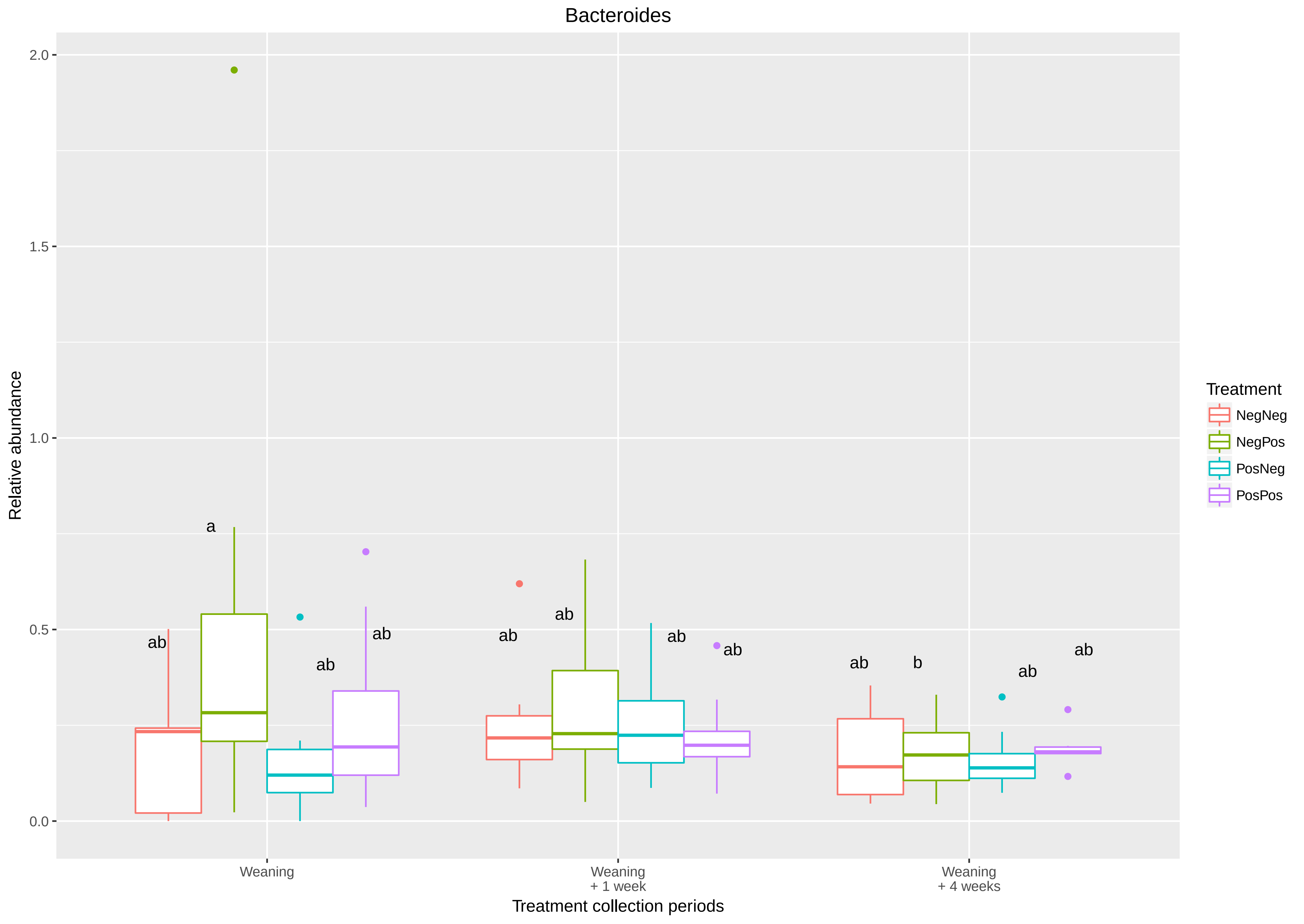
Bacteroides
Relative abundance
2.0
1.5
1.0
0.5
0.0
a
ab
ab
ab
ab
ab
ab
ab
ab
b
ab
ab
Weaning
Weaning
+ 1 week
Weaning
+ 4 weeks
Treatment collection periods
Treatment
NegNeg
NegPos
PosNeg
PosPos

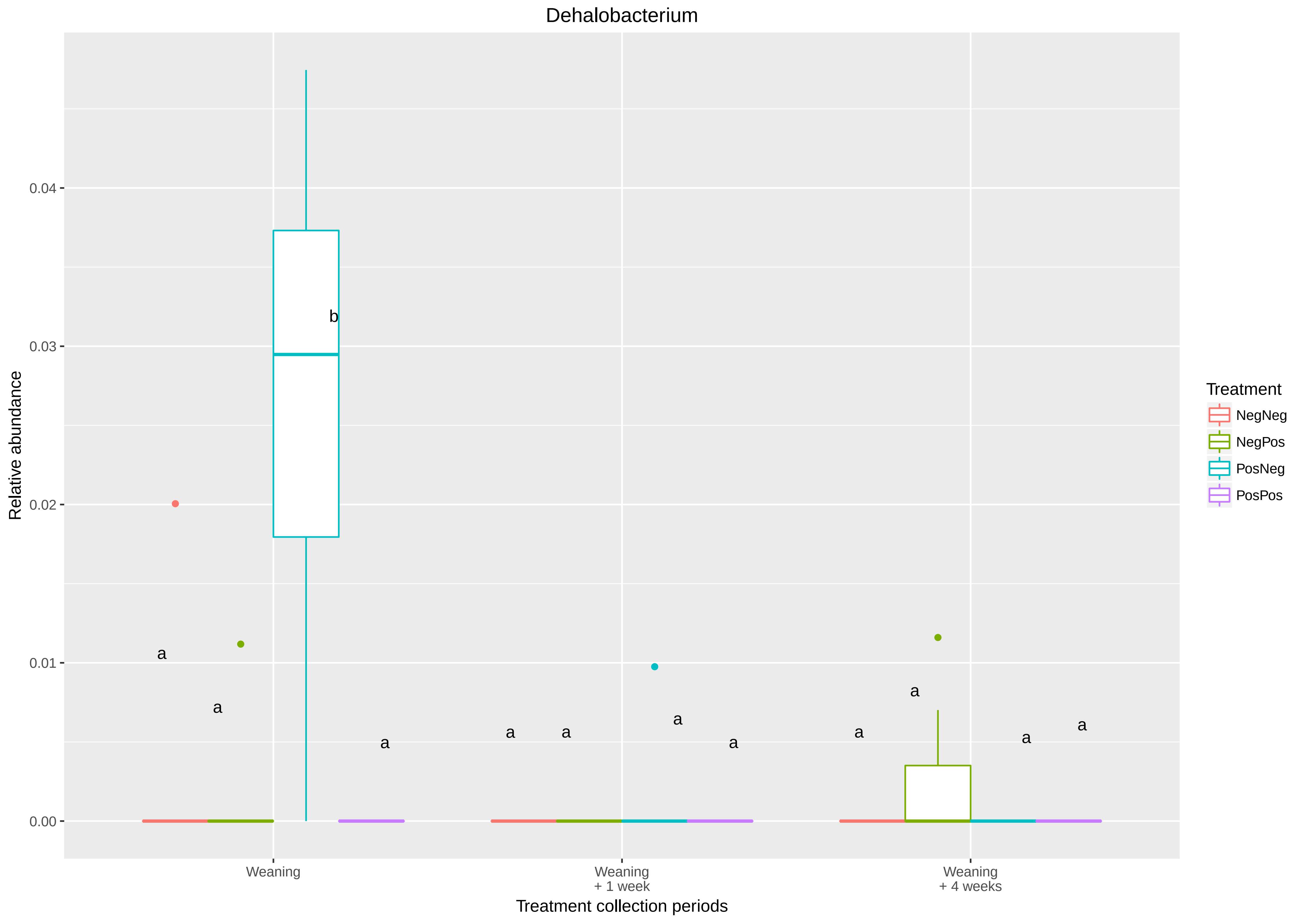
Dehalobacterium
Relative abundance
0.04
0.03
0.02
0.01
0.00
b
a
a
a
a
a
a
a
a
a
a
a
Weaning
Weaning
+ 1 week
Weaning
+ 4 weeks
Treatment collection periods
Treatment
NegNeg
NegPos
PosNeg
PosPos

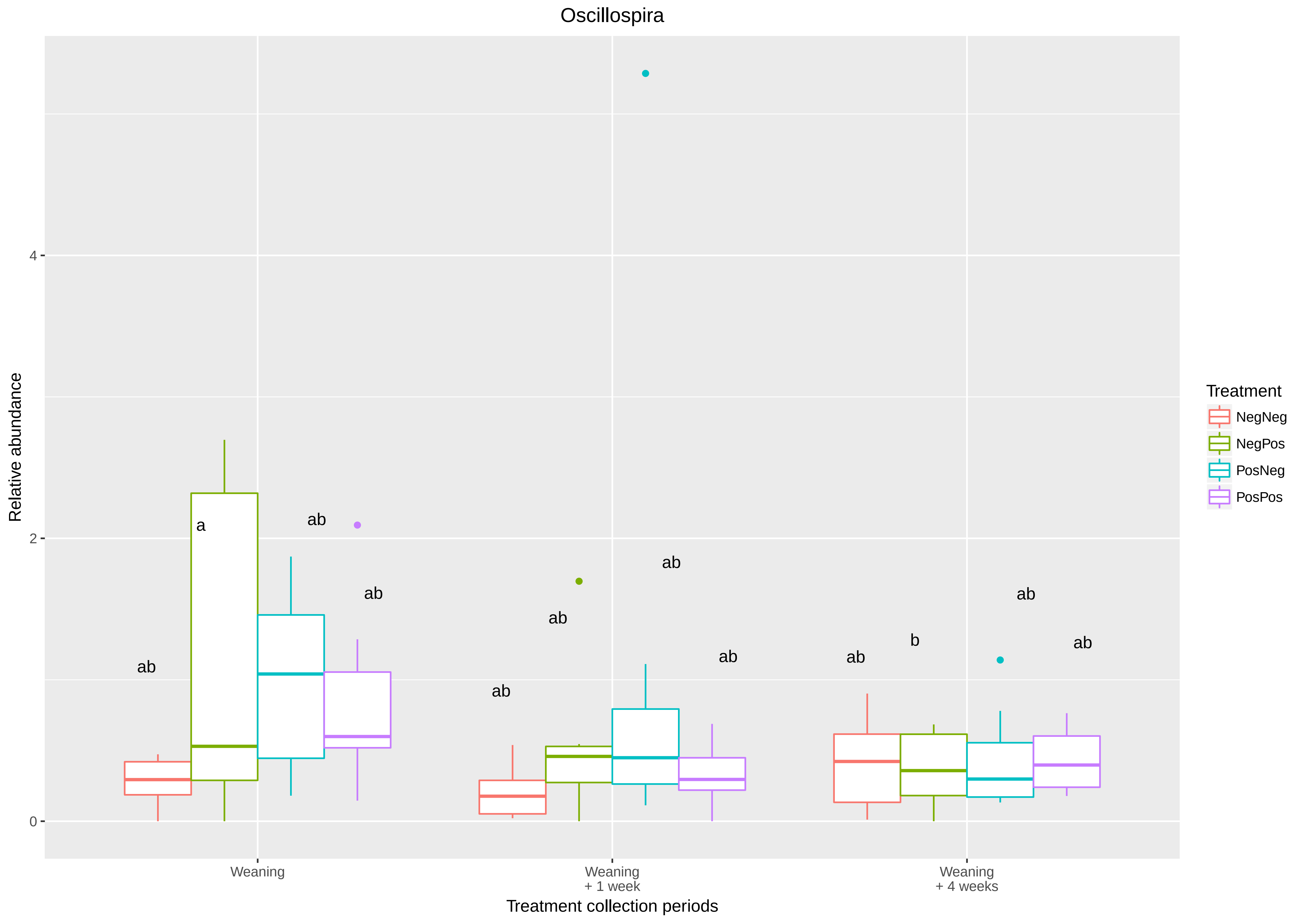
Oscillospira
Relative abundance
0
2
4
a
ab
ab
ab
ab
ab
ab
ab
ab
ab
b
ab
Weaning
Weaning
+ 1 week
Weaning
+ 4 weeks
Treatment collection periods
Treatment
NegNeg
NegPos
PosNeg
PosPos

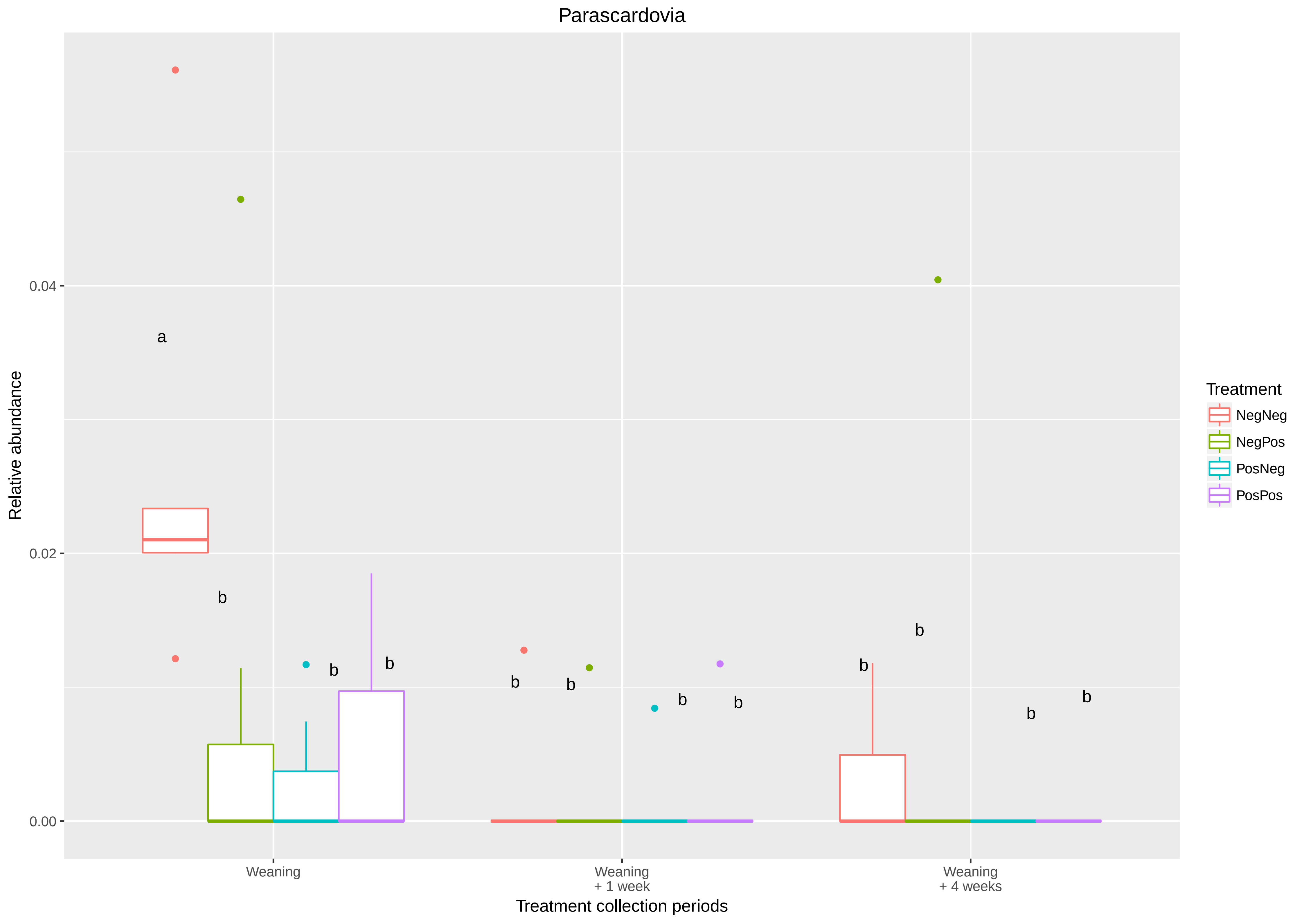

Parascardovia
Relative abundance
0.04
0.02
0.00
a
b
b
b
b
b
b
b
b
b
b
b
Weaning
Weaning
+ 1 week
Weaning
+ 4 weeks
Treatment collection periods
Treatment
NegNeg
NegPos
PosNeg
PosPos

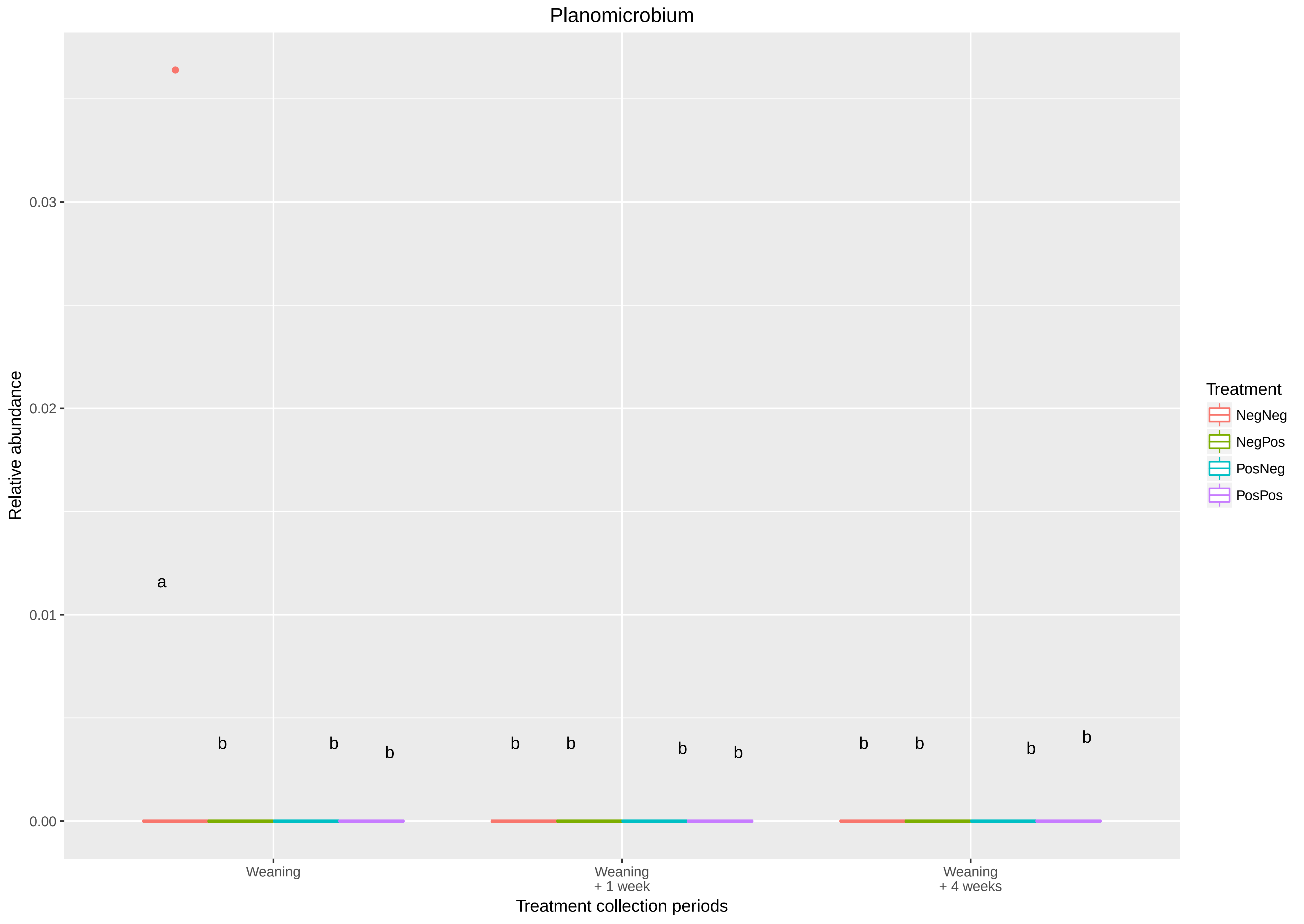
Planomicrobium
Relative abundance
0.03
0.02
0.01
0.00
a
b
b
b
b
b
b
b
b
b
b
b
Weaning
Weaning
+ 1 week
Weaning
+ 4 weeks
Treatment collection periods
Treatment
NegNeg
NegPos
PosNeg
PosPos

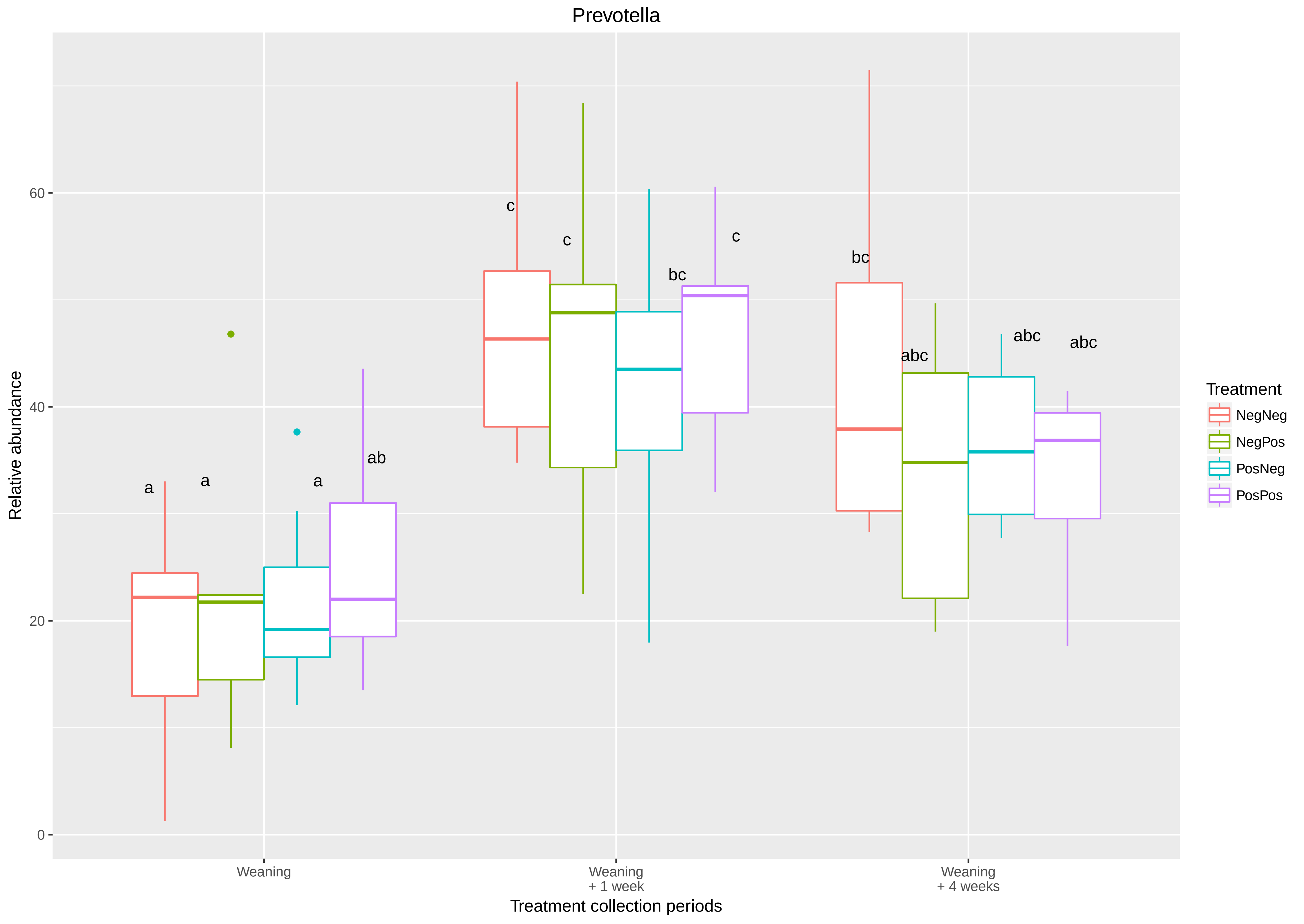

Prevotella
Relative abundance
0
20
40
60
Weaning
Weaning
+ 1 week
Weaning
+ 4 weeks
Treatment collection periods
Treatment
NegNeg
NegPos
PosNeg
PosPos
a
a
a
ab
c
c
bc
c
bc
abc
abc
abc

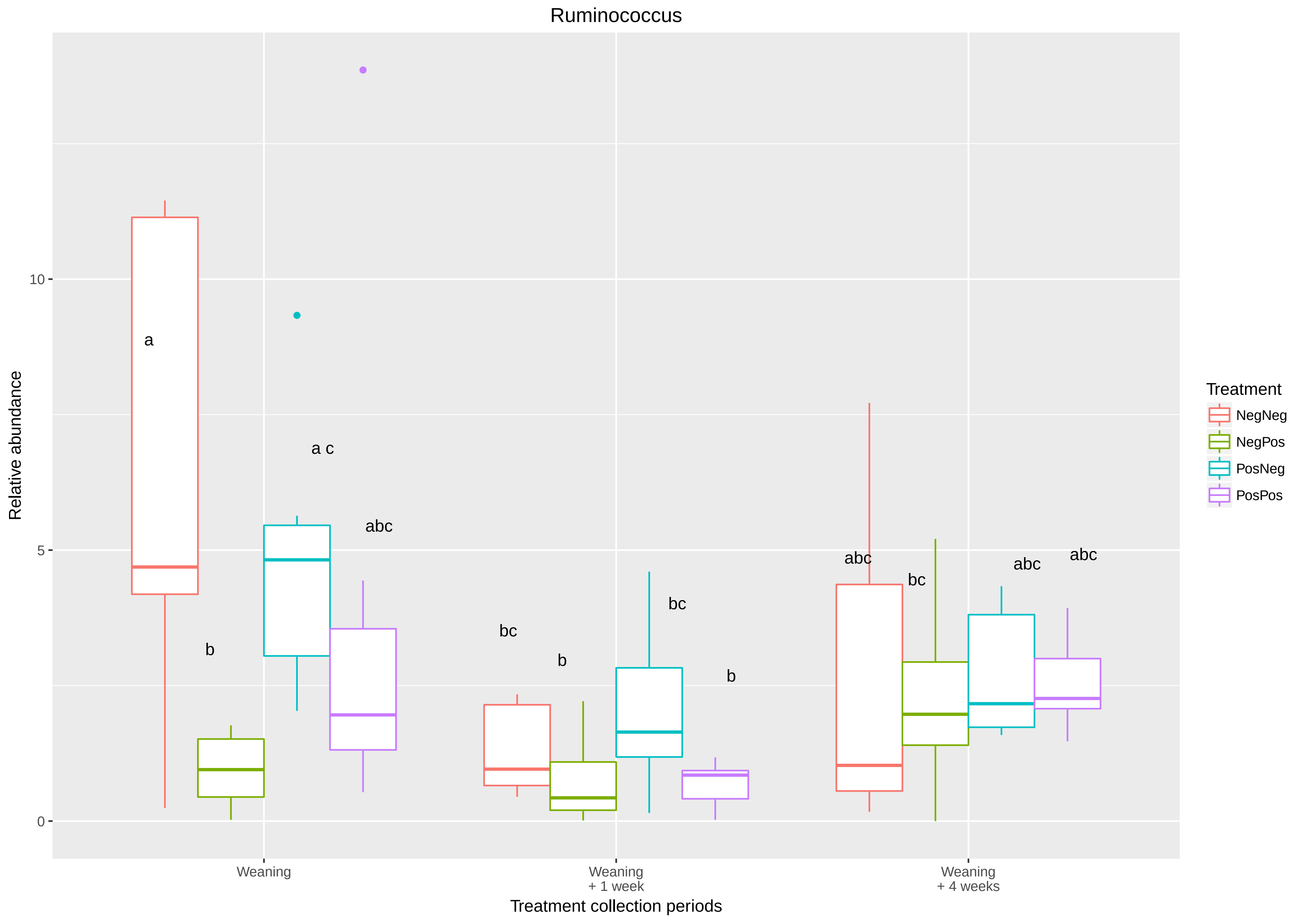
Ruminococcus
Relative abundance
0
5
10
a
b
a c
abc
bc
b
bc
b
abc
bc
abc
abc
Weaning
Weaning
+ 1 week
Weaning
+ 4 weeks
Treatment collection periods
Treatment
NegNeg
NegPos
PosNeg
PosPos

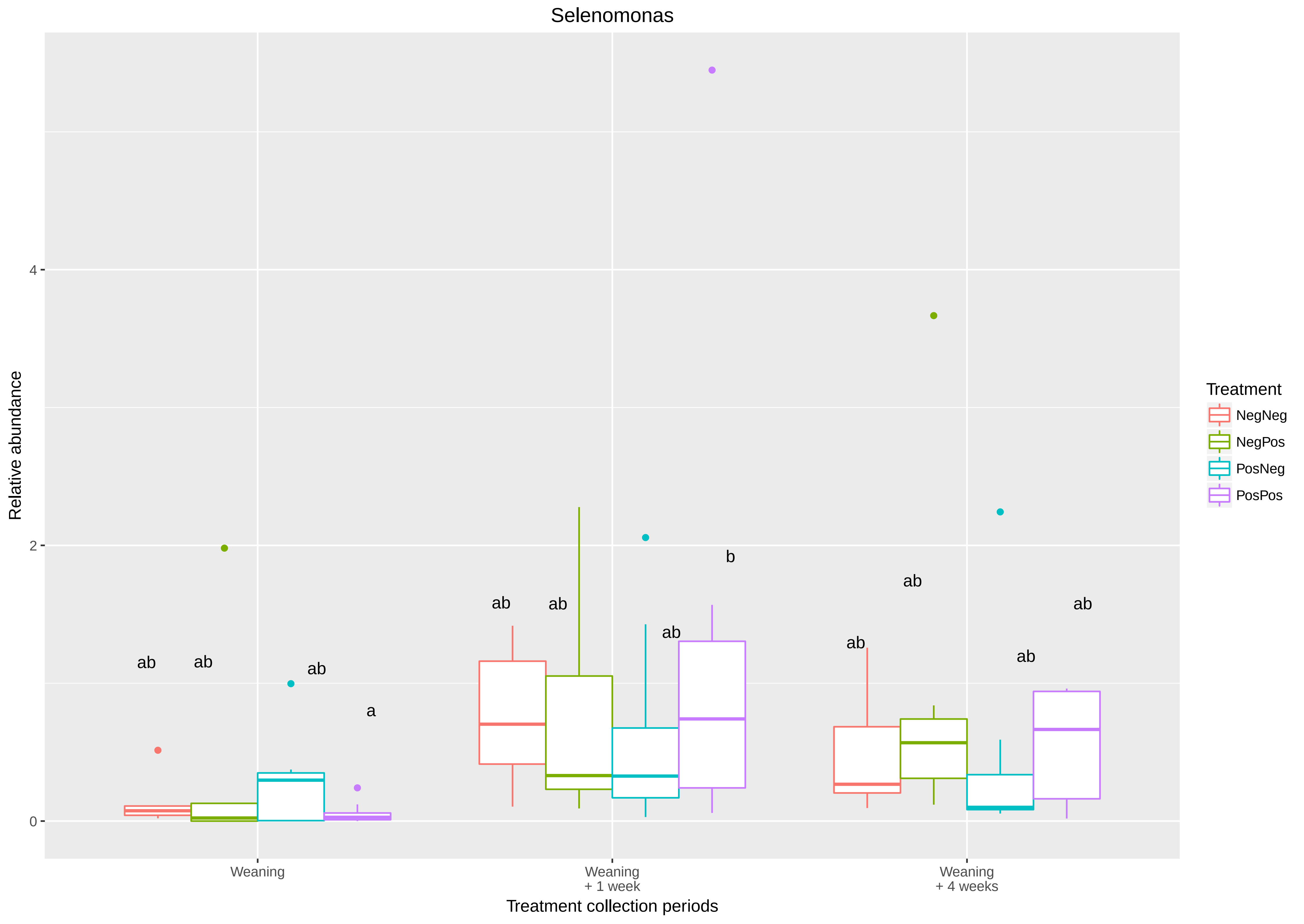

Selenomonas
Relative abundance
0
2
4
ab
ab
ab
a
ab
ab
ab
b
ab
ab
ab
ab
Weaning
Weaning
+ 1 week
Weaning
+ 4 weeks
Treatment collection periods
Treatment
NegNeg
NegPos
PosNeg
PosPos
